# Transcriptional response of *Aspergillus fumigatus* to copper and the role of the Cu chaperones

**DOI:** 10.1080/21505594.2021.1958057

**Published:** 2021-09-01

**Authors:** Duaa Anabosi, Zohar Meir, Yana Shadkchan, Mariana Handelman, Ammar Abou-Kandil, Annie Yap, Daniel Urlings, Morgan S. Gold, Sven Krappmann, Hubertus Haas, Nir Osherov

**Affiliations:** a Department of Clinical Microbiology and Immunology, Sackler School of Medicine Ramat-Aviv, Tel-Aviv, Israel; b Institute for Molecular Biology, Medical University Innsbruck, Austria; c Microbiology Institute, Clinical Microbiology, Immunology and Hygiene University Hospital and Friedrich-Alexander-University (FAU) of Erlangen-Nürnberg, Erlangen, Germany

**Keywords:** *Aspergillus fumigatus*, Cu transcriptome, Cu chaperones, oxidative stress, virulence

## Abstract

*Aspergillus fumigatus* is the leading cause of life-threatening invasive mold infections in immunocompromised individuals. This ubiquitous saprophyte possesses several natural attributes allowing it to evade the immune system, including the ability to withstand high toxic Cu concentrations within the phagosomes of macrophages and neutrophils. We previously established that at high levels, Cu binds and activates the *A. fumigatus* transcription factor AceA, which upregulates the expression of the Cu exporter CrpA to expel excess Cu. Deletion of *aceA* or *crpA* result in extreme Cu sensitivity and attenuated virulence.

To identify other elements participating in resistance to Cu, we performed a genome-wide analysis of the transcriptome by RNAseq to analyze the AceA-dependent response of *A. fumigatus* to excess Cu. We deleted key genes whose transcription was strongly upregulated by high Cu, including those encoding homologs of the three Cu chaperones *cox17, atx1* and *ccs1*. Detailed analysis of these genes indicates that in *A. fumigatus, cox17* is an essential gene with a possible role in respiration, the *atxA* gene product participates in reductive iron uptake and *ccsA* encodes the Cu chaperone activating *A. fumigatus* Sod1. Interestingly, although the *ccsA*-null strain was extremely sensitive to high Cu and oxidative stress, it was not attenuated in virulence in a mouse model of invasive pulmonary aspergillosis.

Our work provides (i) a detailed view of the genome-wide transcriptional response of *A. fumigatus* to excess Cu, (ii) identification of the AceA-dependent transcriptome and (iii) analysis of the roles of the three Cu chaperones *cox17, atxA* and *ccsA.*

## INTRODUCTION

*Aspergillus fumigatus* is a saprophytic mold which is widely found in the environment [1]. *A. fumigatus* is now the most common invasive mold pathogen in developed countries and is the leading cause of death in leukemia and bone marrow transplant patients, estimated to infect at least 200,000 patients/year worldwide [2].

During phagocytosis, pathogenic microbes including *A. fumigatus* encounter high levels of toxic copper (Cu) due to a protective response by the host. Activated macrophages express high levels of the Cu transporter Ctr1p, raising intracellular Cu levels. The Cu-transporting P-type ATPase ATP7Ap is transported from the Golgi to the phagolysosomal membrane, where it pumps high levels of Cu into the phagolysosome, killing the ingested pathogen by generation of reactive oxygen species (ROS) through Fenton chemistry and by displacement of other metal co-factors [3].

The protective response of *A. fumigatus* to high levels of Cu has been recently studied. In response to high extracellular Cu concentrations, *A. fumigatus* transcription factor AceA upregulates the expression of the Cu efflux transporter CrpA [4,5]. Deletion of *aceA* and *crpA* in *A. fumigatus* results in hypersensitivity to both high extracellular Cu and ROS *in vitro*. The corresponding deletion strains accumulate higher Cu levels and are more susceptible to killing by macrophages. They show reduced growth and are less virulent in a mouse model of pulmonary infection [5,6].

Here, to extend our knowledge of the global *A. fumigatus* response to excess Cu, we performed RNAseq analysis. To identify the AceA dependent regulon, the analysis was done on both WT and *aceA*-null congenic strains.

We deleted and characterized several of the genes highest upregulated by Cu. In particular, we focused on the genes encoding the *A. fumigatus* Cu chaperones Cox17, CcsA, and AtxA which have not been previously studied in the filamentous fungi. In the yeast *Saccharomyces cerevisiae*, Cox17p facilitates the delivery of Cu to mitochondria, where it is ultimately incorporated into cytochrome oxidase [7], whereas Ccs1p delivers Cu to copper-containing superoxide dismutase 1 (SOD1) [8]. *S. cerevisiae* Atx1p transports copper to the Ccc2p Cu transporter in the trans-golgi network, where it is then incorporated into Fet3p ferric reductase involved in high-affinity iron uptake and other enzymes [9]. We present the phenotypic characterization of these chaperone-encoding genes in *A. fumigatus*.

## Materials and methods

### Strains and culture conditions

*A. fumigatus* was grown on YAG solid medium (0.5% (w/v) yeast extract, 1% (w/v) glucose, 10 mM MgCl_2_, supplemented with 0.1% (v/v) trace elements solution, and 0.2% (v/v) vitamin mix) and harvested in 0.2% (v/v) Tween-20, resuspended in double distilled water (DDW), and counted with a hemocytometer. Minimal medium (MM) contains 70 mM NaNO3, 1% (w/v) glucose, 12 mM potassium phosphate pH 6.8, 4 mM MgSO4, 7 mM KCl and 0.1% (v/v) trace elements solution, 1.5% (w/v) agar.

The strains used in this work are listed in supplementary Table S1 and their construction and verification is displayed in supplementary Tables S2-S8 and supplementary Figures S1, S3-S9. *A. fumigatus* strain *CEA17* and isogenic *akuB*^KU80^ (WT) [10] were used to generate all mutant strains described in this work (Supplementary Table S1. *A. fumigatus* strain *akuB*^KU80^ was used as the WT control in all experiments.

### Droplet assay

Freshly harvested conidia were serially diluted in sterile water to obtain defined concentrations of 10^6^, 10^5^, 10^4^, and 10^3^ spores/ml. Conidia were spotted in a volume of 10 μl on plates in the presence of the specified stress-inducing agents. Growth was documented after 48 h of incubation at 37°C.

### RNAseq analysis and qPCR verification of select genes

*A. fumigatus* WT and *∆aceA* conidia were inoculated into 50 ml MM liquid medium without copper to a final concentration of 10^6^ spores/ml in twelve 125 ml Erlenmeyer flasks under four conditions: (A) WT strain, grown for 25 h at 37°C, no copper added (samples 1–3), (B) WT strain, grown for 24 h at 37°C with 1 h exposure to 200 µM copper (samples 4–6), (C) *∆aceA* strain, grown for 25 h at 37°C, no copper added (samples 7–9) and (D) *∆aceA* strain, grown for 24 h at 37°C with 1 h exposure to 200 µM copper (samples 10–12). Hyphal mass was filtered and harvested with miracloth, washed, towel dried and lyophilized. Lyophilized material was ground to a powder and total RNA was purified with the Qiagen RNeasy plant Mini kit. RNA quality was verified on RNA gel, NanoDrop 2000 (ThermoFisher Scientific) and TapeStation (System 4150, Agilent) before sequencing. Samples were sent to GATC Biotech AG (Germany) for the Next-generation sequencing section. For the bioinformatics analysis, adapters were trimmed with the cutadapt tool (v1.8) [11]. After adapter removal, reads shorter than 40 nucleotides were discarded (cutadapt option – m 40) and also reads with more than 80% polyA/T were discarded. The *A. fumigatus A1163* genome and annotation were downloaded from http://www.aspergillusgenome.org (version s03-m05-r05). TopHat (v2.0.10) was used to align the reads to the *A. fumigatus A1163* genome [12]. Counting reads on genes (downloaded from *A. fumigatus A1163*) was done with HTSeq-count (version 0.6.1p1) [12,13]. Differential expression analysis was performed with DESeq2 (1.6.3) [14]. Raw P values were adjusted for multiple testing by procedure of Benjamini and Hochberg [15]. An excel file was supplied with the raw and normalized counts, gene expression fold changes for all the genes between the groups, together with their p-values and false discovery rate (FDR) statistical scores associated. Significantly upregulated or downregulated genes were defined as genes with a fold change of above 1.3, or below −1.3, respectively, with adjusted p-value <0.05, and maximal count >20. Gene ontology analysis (AspGD gene ontology term finder on default setting) was used to find enriched pathways/biological processes/functions in the differentially-expressed genes.

For qPCR verification of gene expression of select genes, both *A. fumigatus akuB*^KU80^ (WT) [10] and *∆aceA* strains were inoculated (1x10^6^ conidia) in 25 ml liquid MM containing 1 µM Cu overnight at 37°C and then 200 µM Cu added for another 1 h to the samples exposed to elevated Cu. The mycelium was harvested on miracloth and transferred to liquid nitrogen. The samples were lyophilized. RNA extraction was performed with the RNeasy Mini Kit (QIAGEN®) according to the protocol described in the handbook. The samples were run on an RNA gel to verify they were intact. Then cDNA synthesis was done with the Thermo® verso cDNA synthesis kit. qPCR was performed by mixing 0.25 µM of each primer for each tested gene, 5 µl DDW, 5.5 µl cDNA each – except for the *atxA* mix which contained; 1 µM primers, 3.5 µl DDW, 5.5 µl cDNA). (see qPCR primers and detailed protocol in Supplementary Table. S9). β-tubulin served as control. Statistics were done with Excel.

### *Northern Blot Analysis*-

*A. fumigatus* WT was grown for 24 h at 37°C (1 × 10^6^ conidia/ml) with glutamine as nitrogen source. For limitation of copper, iron or both, supplementation with the respective metal was omitted. Northern analysis was performed as descibed previously [16].

### SOD1 enrichment and activity assay in non-denaturing PAGE

SOD activity analysis was carried out according to Lambou *et al.*, (2010) [17]. 10^8^ spores were suspended in 25 ml YAG liquid medium and incubated with shaking for 16 h at 30°C and 150 rpm. Then mycelium was collected on miracloth, washed twice with DDW, frozen in liquid nitrogen and lyophilized. The lyophilized mycelium was disrupted to powder with glass beads, a 150 µl volume of powder was taken, with addition of 300 µl 20 mM pH 7.8 phosphate buffer supplemented with 3 µl protease inhibitor solution (x100), the mix was then vortexed at 4°C for 2 min. Cell wall removal was done by low speed centrifugation, 300 g at 4°C for 5 min. The supernatant was collected and Bradford analysis of protein concentration performed. The supernatant was used for SOD activity detection. To detect SOD activity, both 8% non-denaturating, non-reducing polyacrylamide running gel, and 4% stacking gel were used. Then, 50 µg of total protein with 3 µl bromphenol blue, were loaded per well. Electrophoresis was carried out at 120 V at room temperature for 2.5 h, followed by staining with the SOD1 substrate to measure activity of the SOD protein. Staining was done based on [18]. After electrophoresis, the gel was washed twice in ice-cold water for 10 min. then soaked in 2.45 mM NBT solution (Sigma) for 40 min in the dark with gentle shaking at 50 rpm. Next, the gel was incubated in 2.8 × 10^−5^ M riboflavin and 0.028 M TEMED in 0.036 M potassium phosphate buffer pH 7.8 for 30 min in the dark. Then, the gel was illuminated on a white light box for 5–15 min. Upon illumination, an achromatic band indicating zones of activity appeared in the region of the gel where SOD proteins were present.

### Mouse model of invasive pulmonary aspergillosis

Six-week-old ICR female mice were immunocompromised by two subcutaneous injections with 300 mg/kg cortisone acetate, given 3 d before infection, and on the day of infection. The mice were anaesthetized by intraperitoneal (IP) injection with 100/10 mg/kg ketamine + xylazine and infected intranasally with 5 × 10^5^ dormant spores/mouse, suspended in 20 µl of 0.2% Tween 20 in saline solution (0.9% w/v NaCl) (10 µl in each nostril). Endpoints for sacrifice included a drop of >15% in body weight or signs of acute distress. Mice were monitored for 21 d. Results were analyzed with the log-rank test for Kaplan–Meyer survival curves in GraphPad Prism software. Animal studies were done in accordance with Tel-Aviv University institutional policies. All efforts were made to minimize the number of animals used and animal suffering.

### Histopathology

Mice were sacrificed 2 d after infection and their lungs were removed and sent for histological staining with Grocott’s methenamine silver stain (GMS; fungal staining) and hematoxylin and eosin (H&E; tissue and nuclear staining). Microscopic analysis was performed with an Olympus AX-70 microscope at 20X magnification. Images were recorded on an Olympus DP72 camera.

### Measurement of fungal burden

Quantification of viable *A. fumigatus* cells within the lungs of infected mice was implemented by colony forming unit (CFU) counts. At 48 h post-infection, mice were euthanized, lungs were removed aseptically and homogenized in 2 ml of sterile PBS with a tissue homogenizer (TissueRuptor, Qiagen, Hilden, Germany). Duplicates of serial dilutions of the tissue homogenates were plated onto Sabouraud plates with antibiotics. The plates were incubated for 24 h and CFUs were calculated per lung homogenate. Only *A. fumigatus* colonies were observed on the plates.

### Statistical analysis

Data and statistical analysis were analyzed with the GraphPad Prism 8 software package (GraphPad Software, Inc, San Diego, CA, USA) or the Microsoft Excel software package (Microsoft Corporation, Redmond, WA, USA). The ANOVA test was used for significance testing of two groups. Differences between the groups were considered significant at *P* ≤ 0.05. Mortality results were analyzed by the log-rank test for Kaplan–Meyer survival curve, but t test was used for significance testing of two groups. Differences between the groups were considered significant at *P* ≤ 0.05.

## RESULTS

### Transcriptome analysis of the response of WT and ΔAceA to excess Cu

*A. fumigatus* wild-type (WT; A1163) and congenic *∆aceA* conidia were germinated in MM without Cu for 25 h at 37°C and treated with excess (200 µM) Cu for 1 h. The mycelium was harvested, total RNA prepared and analyzed by RNAseq as described in the methods.

### Differentially expressed genes in the WT strain when exposed to high Cu

Differential expression analysis revealed 2,400 genes up regulated and 2,662 genes down regulated in the WT strain when exposed to high Cu. Of these, 4,774 (2,264 upregulated and 2,510 downregulated) annotated genes were evaluated by GO term enrichment analysis.

**Figure 1.**
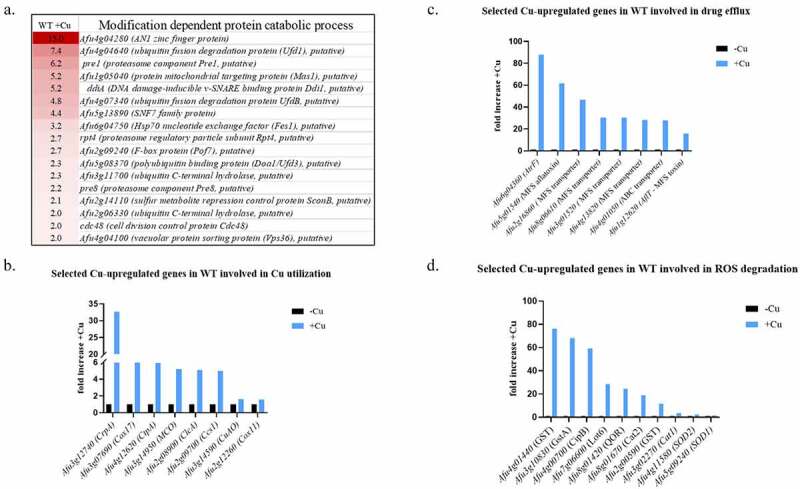
Differentially expressed genes in the WT *A. fumigatus* strain when exposed to high Cu (200 µM) for 1 h. (a) Genes involved in modification-dependent protein catabolic process were significantly up-regulated by Cu (*P*-value = 0.00452). Selected Cu-upregulated genes involved in (b) Cu-utilization, (c) drug efflux and (d) ROS degradation

Gene categories significantly up-regulated by Cu includes 49 genes involved in the ubiquitin dependent breakdown of proteins (P-value = 0.00452) (Figure 1a and supplementary Table S10).

Three groups of upregulated genes are of particular interest because of their possible protective roles: (i) genes encoding proteins participating in Cu-utilization, including as previously established, the *crpA* Cu exporter [6], the intracellular *ctpA* Cu transporter and the two Cu chaperones encoded by *cox17* (*Afu3g07690*) and *ccsA* (*Afu2g09700*) (Figure 1b). (ii) genes encoding efflux transporters. They include *atrF* (*Afu6g04360*) – encoding a previously described ABC drug exporter involved in azole resistance [19] and six MFS transporters (Figure 1c).

(iii) genes participating in the breakdown of ROS presumably generated by Cu, such as catalases, glutathione reductases and oxidoreductases (Figure 1d). Examples include *cipB* encoding a zinc-binding oxidoreductase (*Afu4g00700*), two superoxide dismutases *sod1(Afu5g09240*) and *sod2 (Afu4g11580)* [17], and two glutathione S-transferases, two catalases, *cat1 (Afu3g02270)* encoding a mycelial catalase and *cat2* (*Afu8g01670*) – encoding a bifunctional mycelial-expressed catalase-peroxidase. Deletion of both genes resulted in complete loss of catalase activity and reduced virulence [20]. Another interesting gene which is most strongly upregulated by Cu is the nitrogen metabolite repressor *nmrA* (*Afu7g06920*) upregulated 2231 fold.

Significantly enriched down-regulated gene categories includes ribosome biogenesis (P-value = 1.3X10^−17^), ribonucleoprotein complex biogenesis (P-value = 9.46X10^−14^) and rRNA processing (P-value = 1.62X10^−9^) [Table S10B]. This is likely because Cu-induced cell stress inhibits protein synthesis to conserve energy.

Of interest are downregulated genes taking part in Cu-uptake, including *ctrA2* (*Afu6g02810*) – encoding a Cu transporter, downregulated by Cu 4.85 fold and *ctrA1* (*Afu3g13660*) – encoding a second Cu transporter family protein of undefined function, downregulated by Cu 2.74 fold. CtrA2 is a high affinity Cu importer which is expressed to ensure Cu sufficiency under conditions where Cu is limited [21]. Downregulation of these genes is likely an attempt by the fungus to reduce the uptake of toxic Cu under the stress conditions we tested.

### Differentially expressed genes in the WT strain vs. the ∆aceA strain when exposed to high Cu

Cu treatment significantly increased the expression of 230 genes in the WT strain and not in the *∆aceA* strain, indicating that AceA directly or indirectly activates these genes. The expression of 725 genes was decreased in the WT and not in the *∆aceA* strain when exposed to high Cu, suggesting that AceA directly or indirectly inhibits these genes. Out of these, 890 (213 upregulated and 677 downregulated) annotated genes were evaluated by GO term enrichment analysis.

**Figure 2.**
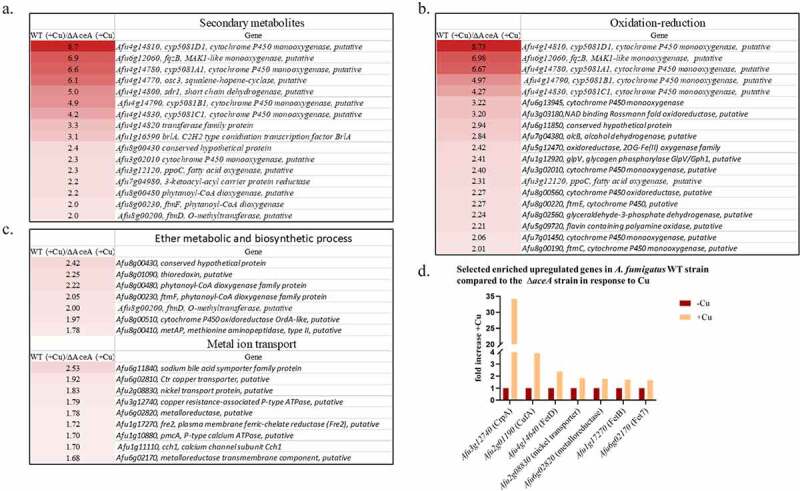
Differentially expressed genes in the WT *A. fumigatus* strain vs. the *∆aceA* strain when exposed to high Cu (200 µM) for 1 h. Genes involved in (a) secondary metabolite biosynthesis, (b) oxidation-reduction (c) ether metabolic and biosynthetic processes, metal ion transport and (d) Cu, Ni and Fe transport, were significantly more strongly expressed in the WT vs. the *∆aceA* strain (*P*-value < 0.05)

Genes participating in secondary metabolite biosynthesis were significantly enriched following exposure of the WT vs. *∆aceA* strain to high Cu levels (*P*-value = 3.18 × 10^−5^) including the helvolic acid, fumitremorgin and fumagillin biosynthesis clusters (Figure 2a) ether metabolic processes (*P*-value = 8.4 × 10^−4^), metal ion transport (*P*-value = 0.027) and oxidation-reduction (*P*-value = 6.9 × 10^−4^) (Figure 2b, c and Supplementary Table S11A). This suggests that AceA directly or indirectly activates these genes in response to Cu stress.

Genes involved in the transport or binding of Cu and other metals were more strongly upregulated in the WT compared to the *∆aceA* strain (Figure 2d], including *crpA* upregulated 34.18 fold as previously described [6], *cufA* Cu binding transcription factor [6] upregulated 3.9 fold, *fetD* (*Afu4g14640*) – encoding a putative low-affinity iron transporter involved in accumulating iron and potentially zinc during iron starvation [22], upregulated 2.37 fold and three metalloreductases; *freB* participating in reductive iron uptake [23], and the uncharacterized *fre7* and *Afu6g02820*.

Genes involved in neutral amino acid transport (*P*-value = 0.00189) and transmembrane transport (*P*-value = 0.02407) were significantly downregulated in the WT compared to the *∆aceA* strain [Supplemental Table S11B]. Worthy of mention is the putative Cu chaperone-encoding gene *atxA* (*Afu1g08880*) – downregulated by 271 fold in the WT compared with the *∆aceA* strain, implying that AceA strongly suppresses its expression under Cu excess.

**Figure 3.**
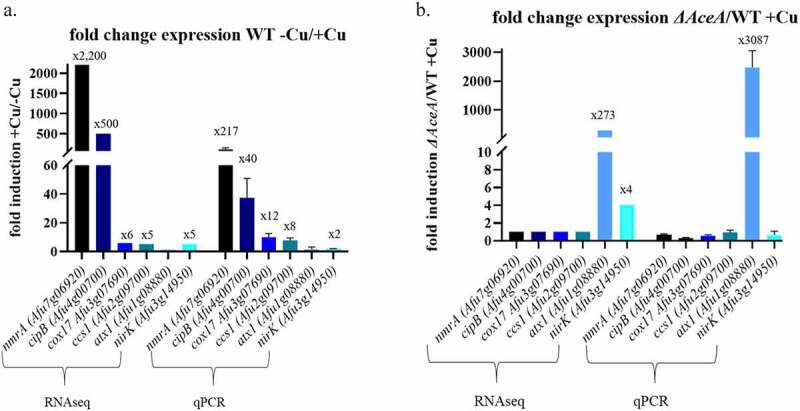
**Expression of putative Cu-associated genes, selected for deletion analysis**. Fold-change expression of Cu-associated genes selected for deletion analysis following exposure to 200 µM Cu for 1 h in (a) WT (treated vs. untreated) and (b) WT vs. *∆aceA* (treated). Results are shown for RNAseq (left) and qPCR verification (right)

After identifying the *A. fumigatus* Cu regulon and its AceA-dependent components by RNAseq, we prioritized six genes for further study and verified their RNAseq results by qPCR (Figure 3). These genes were chosen because of (i) their connection to Cu metabolism based on the literature or (ii) their significantly high induction after exposure to high Cu levels. To test their involvement in Cu homeostasis, we generated deletion strains for these genes and conducted functional analysis experiments under Cu excess and oxidative stress. The selected genes were *Afu7g06920* (*nmrA* redox sensor), *Afu4g00700* (*cipB*, oxidoreductase), *Afu3g07690 (cox17), Afu2g09700 (ccsA), Afu1g08880 (atxA)*, and *Afu3g14950* (*nirK*). The *A. fumigatus* genome contains only three Cu-chaperones *Afu1g08880 (atxA), Afu3g07690 (cox17)*, and *Afu2g09700 (ccsA)* and we hypothesized that they could be involved in protecting against the deleterious effects of excess Cu. If so, we expected that deleting them might generate a mutant displaying a Cu-sensitive phenotype similar to that of the *aceA* and *crpA* deletion mutants.

### Generation of the gene deletion mutants and their phenotypic characterization

The genes listed in Figure 3, henceforth named *nmrA, cipB, cox17, ccsA, atxA* and *nirK*, were deleted as described in the materials and methods. Transformants were verified for gene deletion as described in the Methods section. Heterokaryon analysis of primary transformants showed that the Δ*cox17* deletants grew as tiny aconidial colonies under hygromycin selection, and we were unable to propagate them further (supplementary Fig. S2A), suggesting that *cox17* is an essential gene. Interestingly, as previously demonstrated in *S. cerevisiae*, growth of the *Δcox17* strain was slightly but significantly increased at high levels of Cu (supplementary Fig. S2B).

**Figure 4.**
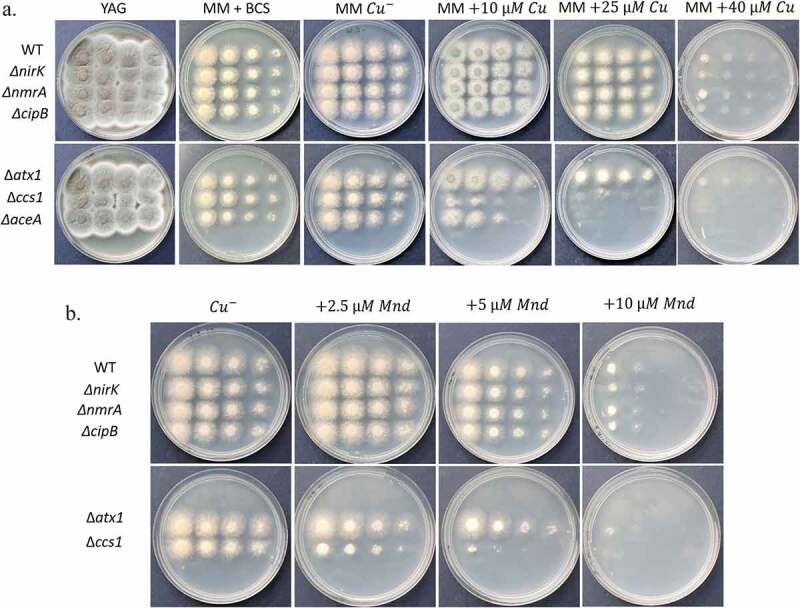
Growth and phenotypes of Cu-induced gene deletion mutants *ΔnirK, ΔnmrA, ΔcipB, ΔatxA* and *ΔccsA*. Strains were point inoculated at 10-fold dilutions on (a) YAG and MM agar plates under Cu starvation and increasing Cu concentrations or (b) MM agar plates under increasing concentrations of menadione to induce oxidative stress. Plates were incubated for 48 h at 37°C

To test for Cu-sensitivity, we point inoculated serial dilutions of the Δ*nirK*, Δ*nmrA*, Δ*cipB*, Δ*atxA* and Δ*ccsA* deletion strains on defined MM agar plates under increasing Cu concentrations. MM containing the Cu chelator bathocuproine disulfonate (BCS) was used to test growth under Cu-depletion. Growth was compared to the WT congenic strain and the previously described Δ*aceA* mutant which is sensitive to excess Cu [6]. We found that the Δ*nirK*, Δ*nmrA*, Δ*cipB* and Δ*atxA* mutants grew normally under Cu-excess or Cu-starvation (Figure 4a). Interestingly, Δ*ccsA* on MM was highly sensitive to Cu and grew at a rate similar to WT only under Cu chelation with BCS or in the absence of Cu (Figure 4a). Δ*ccsA* was slightly less Cu-sensitive than the Δ*aceA* strain. High Cu also generates oxygen radicals and oxidative stressors through the Fenton reaction [4]. We therefore tested the ability of the mutant strains to grow under oxidative stress induced by menadione (Mnd) (Figure 4b). Results indicate that the Δ*ccsA* mutant is sensitive to this compound.

### AtxA participates in reductive iron uptake

**Figure 5.**
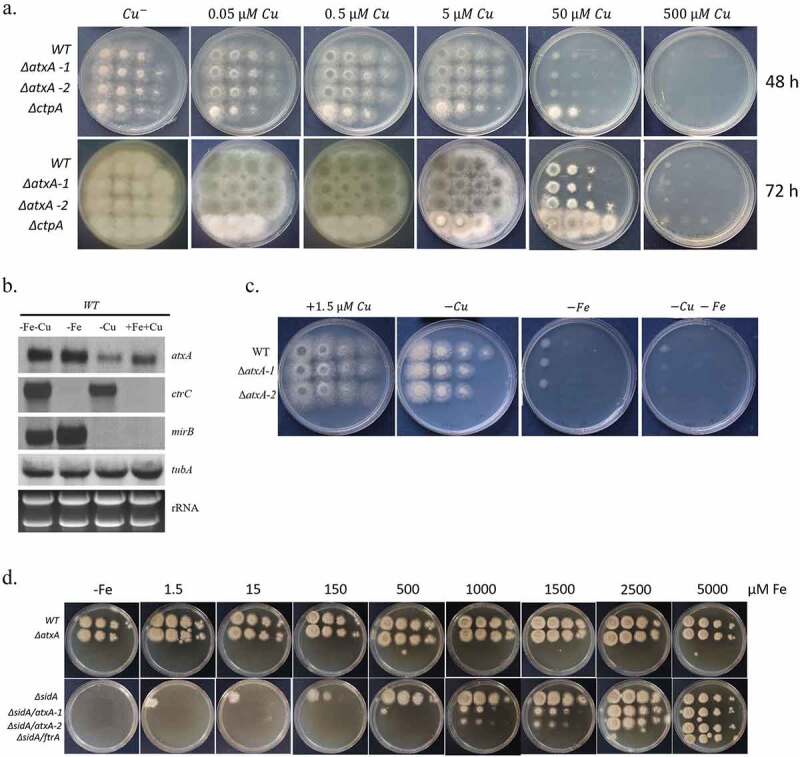
***A. fumigatus* AtxA participates in reductive iron uptake**. (a) WT, two isolates of *ΔatxA* and the *ΔctpA* strain were point inoculated at 10-fold dilutions on MM agar plates for 48 h at 37°C under increasing Cu concentrations. WT, and *ΔatxA* retained conidial melanin pigmentation whereas *ΔctpA* did not. (b) Northern blot of *atxA, ctrC* and *mirB* gene expression in WT under standard conditions or exposed to starvation for Cu (-Cu), Fe (-Fe) starvation or both (-Fe-Cu). *TubA* housekeeping gene expression level and ethidium bromide-stained ribosomal RNA are shown as control for RNA loading and quality. (c) WT and two isolates of *ΔatxA* were point inoculated at 10-fold dilutions on MM agar plates for 48 h at 37°C under Cu starvation (-Cu + 50 µM BCS), Fe starvation (-Fe + 100 µM BPS and 10 mM Ferrozine) or both (-Cu–Fe + 50 µM BCS, 100 µM BPS and 10 mM Ferrozine). (d) Indicated strains were point inoculated at 10-fold dilutions on MM agar plates without Cu under increasing Fe concentrations for 48 h at 37°C

In *Cryptococcus neoformans*, Atx1p chaperones Cu to the Cu transporter Ccc2 for insertion into laccase which produces melanin [24]. In *A. fumigatus*, melanin is synthesized by the laccases Abr1 and Abr2, supplied with Cu by the copper transporter CtpA, the homolog of Ccc2, located in intracellular vesicles [25]. We hypothesized that *A. fumigatus* AtxA chaperones Cu to CtpA to facilitate conidial melanization. To test this, we grew WT, Δ*atxA* and Δ*ctpA* strains on plates containing increasing Cu levels and analyzed conidial color after 2 d of growth (Figure 5a). We assumed that if AtxA and CtpA act in the same pathway, they would display a similar level of melanization only at relatively high Cu levels. Without Cu, all the strains produced white conidia. Melanization in the WT and Δ*atxA* strains began at 0.05 µM Cu and increased to maximal levels at 0.5 µM Cu. In contrast, the Δ*ctpA* mutant began producing melanized green conidia only after 3 d of growth at 50 µM Cu (Figure 5a-lower image). This result demonstrates that deletion of *atxA* does not phenocopy deletion of *ctpA* and consequently, it is unlikely that AtxA is the main Cu chaperone for CtpA.

In *S. cerevisiae*, Atx1p chaperones Cu to the copper transporter Ccc2p for insertion into Fet3p, which is a multicopper oxidase required for high-affinity reductive iron uptake. *Atx1* deletion in *S. cerevisiae* leads to an inability to take up iron [9]. We reasoned that if *A. fumigatus* AtxA participates in iron uptake, transcription of its corresponding gene will be upregulated under iron starvation. To test this, we performed Northern blot analysis of WT grown on MM in the presence or absence of Fe and/or Cu (Figure 5b and independently repeated experiment in supplementary, Fig. S10). *AtxA* expression increased under iron starvation irrespective of Cu levels, indicating a possible connection between *atxA* and iron uptake. Controls included *ctrC* (Cu importer) and *mirB* (siderophore uptake transporter) expression, which as expected, increased under Cu and Fe starvation, respectively. We next tested if the *A. fumigatus* Δ*atxA* mutant is sensitive to iron insufficiency in the presence of iron chelators (100 µM bathophenantroline/BPS and 10 mM ferrozine; MM-Fe) (Figure 5c). The Δ*atxA* mutant was not more sensitive than WT under iron insufficiency, but showed a very slight increase in sensitivity under iron and Cu insufficiency (MM-Cu-Fe). However, unlike in yeast, *A. fumigatus* can also take up iron with secreted siderophores, which can bypass high-affinity reductive iron uptake [26]. For this reason, we generated a double deletion *ΔsidA/ΔatxA* mutant in which siderophore biosynthesis is inactivated. We hypothesized that if reductive iron uptake depends on Cu supplied by AtxA, then the *ΔsidA/ΔatxA* strain would phenocopy the *ΔsidA/ΔftrA* mutant, which lacks both siderophore-dependent and reductive iron uptake system, and thus grows only at extremely high (5 mM) Fe concentrations. Under Cu insufficiency, *ΔsidA/ΔatxA* isolates grew more poorly than either the *ΔsidA* or *ΔatxA* single mutants (Figure 5d), suggesting that in the absence of siderophore iron uptake, AtxA participates in reductive iron uptake, most likely by transferring Cu to FetC to activate it. However, AtxA is apparently not the only source of Cu for this system, because the *ΔsidA/ΔftrA* mutant was much more dependent on high levels of added Fe (5 mM), compared to *ΔsidA/ΔatxA* (0.5–1 mM Fe) (Figure 5d).

### The ΔccsA mutant phenocopies the Δsod1 null in A. fumigatus

**Figure 6.**
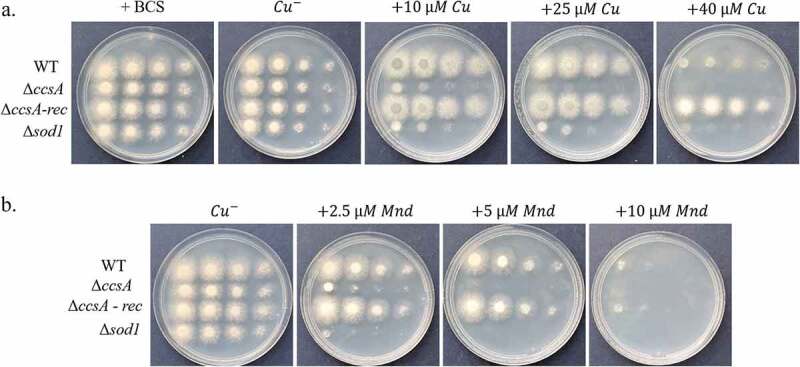
Deletion of *A. fumigatus ccsA* phenocopies deletion of *sod1*. (a) WT, *ΔccsA*, reconstituted *ΔccsA-rec* and *Δsod1* strains were point inoculated at 10-fold dilutions on (A) MM agar plates under Cu starvation (+50 µM BCS, -Cu) and increasing Cu concentrations and (b) MM agar plates under increasing concentrations of menadione to induce oxidative stress. Plates were incubated for 48 h at 37°C

In *S. cerevisiae*, Ccs1p chaperones Cu to the Cu/Zn superoxide dismutase Sod1p, and *ccs1* deletion leads to sensitivity to oxidative stress [27]. We therefore tested if likewise, the *A. fumigatus* Δ*ccsA* mutant is sensitive to high Cu and to oxidative stress exerted by menadione. We inoculated serial dilutions of the WT, Δ*ccsA*, Δ*ccsA-rec* (reconstituted) and Δ*sod1* strains on defined MM agar plates under increasing Cu (Figure 6a) or menadione (Mnd) (Figure 6b) concentrations. Results indicate that the Δ*ccsA* mutant phenocopied the *Δsod1* null in *A. fumigatus* and was highly sensitive to both Cu and menadione. WT growth and Cu and menadione resistance was restored in the reconstituted Δ*ccsA-rec* strain.

### Deletion of *ccsA* blocks *sod1* activity in *A. fumigatus*

**Figure 7.**
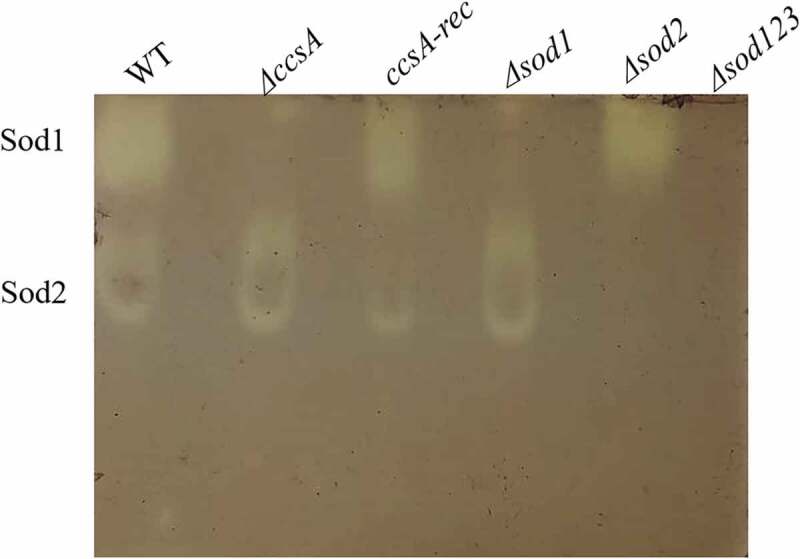
**Deletion of*****A. fumigatus******ccsA* results in loss of sod1 activity**. Protein extracts from WT, *ΔccsA*, reconstituted *ΔccsA-rec, Δsod1, Δsod2, Δsod123* strains were run on a non-denaturing non-reducing polyacrylamide gel. Sod (superoxide dismutase) activity was visualized by NBT reduction within the gel

In *S. cerevisiae, ccs1* deletion leads to loss of Sod1p activity in substrate non-denaturing gel assays [27]. To test this in *A. fumigatus*, protein extracts were prepared from the WT, Δ*ccsA*, Δ*ccsA-rec* (reconstituted) Δ*sod1*, Δ*sod2* and Δ*sod123* strains, run on a non-denaturing non-reducing polyacrylamide gel and SOD activity was visualized by the reduction of NBT (see materials and methods). Results demonstrate that the Δ*ccsA* mutant lacked Sod1 activity, which was restored in the reconstituted Δ*ccsA-rec* strain (Figure 7). Taken together, our results suggest that *A. fumigatus ccsA* transfers Cu to Sod1, thereby activating it. Activated Sod1 provides protection against oxidative stress by neutralizing highly reactive superoxide anions.

### Deletion of *ccsA* does not affect *A. fumigatus* virulence

**Figure 8.**
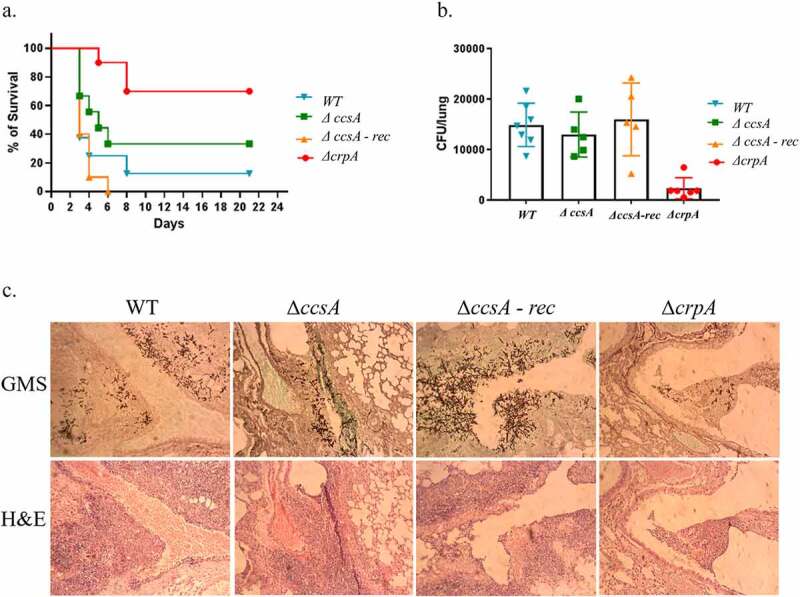
**Deletion of *A. fumigatus ccsA* does not affect virulence**. Cortisone-acetate immunocompromised mice were infected intranasally with WT, *ΔccsA*, reconstituted *ΔccsA-rec* and *ΔcrpA* (attenuated virulence control) conidia. (a) Survival plot of mice during 21 d of infection. (b) Lung fungal load after 48 h of infection. (c) Histopathology of infected lungs after staining with GMS (Gomori methenamine silver, stains hyphae black) or H&E (Hematoxylin and eosin, stains granulocytes purple)

We previously established that deletion of the Cu exporter CrpA leads to Cu sensitivity and reduced virulence in infected mice [6]. Because the *ΔccsA* mutant is even more sensitive to Cu, we reasoned it would also display reduced virulence in mice. To test this, we used a pulmonary model of infection in immunocompromised mice. Four groups of 10 mice were infected with WT, *ΔccsA* null mutant, *ΔccsA-rec* and *ΔcrpA* strains, respectively. Mice were immunocompromised and infected intranasally with *A. fumigatus* spores as described in the methods. We followed mouse survival for 21 d. Surprisingly, the survival curve shows no difference in virulence between the *ΔccsA* null strains compared to the WT (*P* = 0.3479) and *ΔccsA-rec* (*P* = 0.2935) strains (Figure 8a). Control *ΔcrpA*, as expected, exhibited less virulence compared with the WT (*P* = 0.0026**). This result indicates that CcsA does not affect the virulence of *A. fumigatus*, despite the strong *in vitro* sensitivity of the *ΔccsA* null mutant to Cu and oxidative stress. To detect if lung fungal load was reduced in the *ΔccsA* null strain, mice were infected as described above and sacrificed after 48 h. One of their lungs was homogenized and aliquots spread on SAB plates with antibiotics. CFU counts after incubation show no statistically significant differences in fungal load from lungs of mice infected with *ΔccsA, ΔccsA-rec* and WT strains (Figure 8b). As we have previously presented, the fungal load of mice infected with *ΔcrpA* was significantly lower than the WT (*P* = 0.0004***). Lung sections were also analyzed by histopathology, and stained with H&E and GMS. The slides were observed microscopically for fungal lesions (Figure 8c). The lungs of mice infected with *ΔccsA* contained many large fungal lesions similar to mice infected with WT and *ΔccsA-rec* strains. Mice infected with *ΔcrpA* had very few fungal lesions compared with WT. In summary, deletion of *ccsA* does not affect virulence, fungal load and histopathology in a murine model of *A. fumigatus* lung infection.

## DISCUSSION

This study maps the transcriptional changes that occur in WT and *AceA* null *A. fumigatus* after exposure to high levels of Cu. It analyzes the cellular function of key upregulated genes and in particular, those encoding the *A. fumigatus* Cu chaperones AtxA, CcsA and Cox17.

### Analysis of the *A. fumigatus* Cu transcriptome

**Figure 9.**
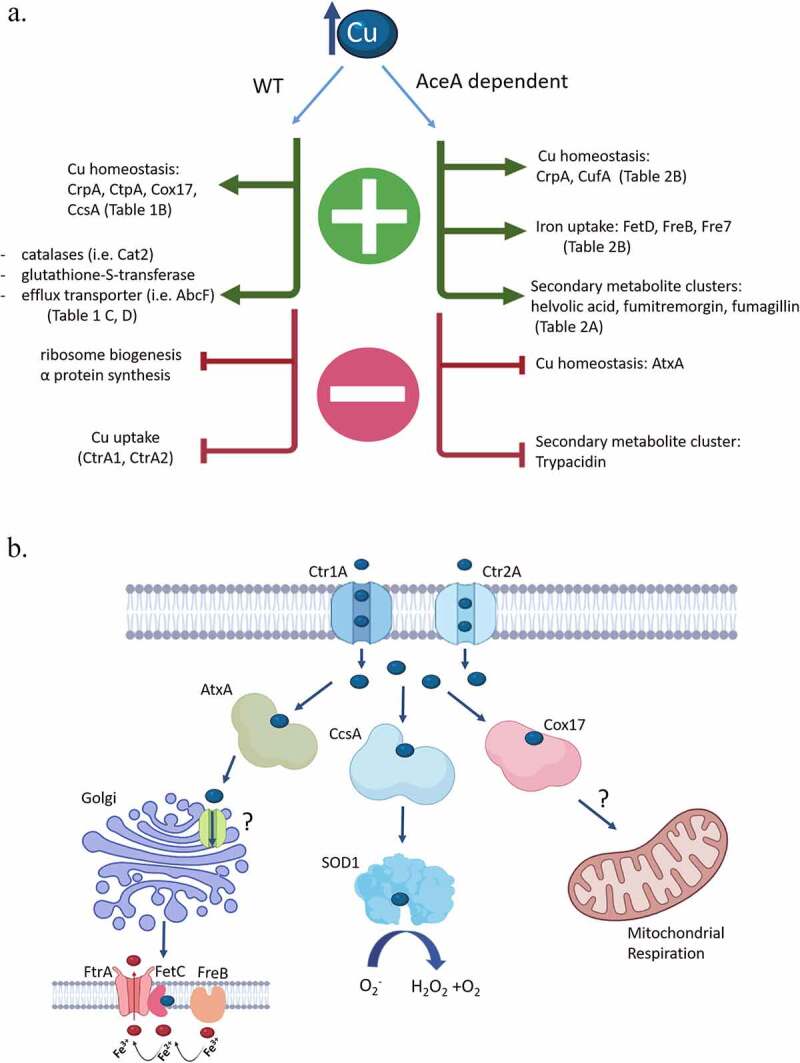
**Summary scheme**. (a) *A. fumigatus* transcriptional response to high Cu concentration. AceA-dependent and independent changes occur in the expression of genes participating in Cu and Fe homeostasis, ROS breakdown and secondary metabolite biosynthesis. (b) Proposed roles of the *A. fumigatus* Cu chaperones Cox17, CcsA and AtxA. *Cox17* is an essential gene containing a mitochondrial localization motif and is probably involved in respiration. CcsA transfers Cu to Sod1 to activate its catalytic activity. AtxA takes part in reductive iron assimilation, possibly by transferring Cu to an unknown ER Cu transporter to activate FetC

The global transcriptional response to excess Cu has been analyzed in *S. cerevisiae* [28], *C. neoformans* [29], *C. albicans* [30] and *Aspergillus nidulans* [31]. We describe for the first time the transcriptome of WT and *AceA* null *A. fumigatus* under Cu stress. In overview (Figure 6a, left), WT *A. fumigatus* exposed to high Cu responds by upregulating expression of Cu exporter (CrpA), Cu chaperones (Cox17, CcsA), ROS detoxifying enzymes (Catalases, glutathione-S-transferases), and efflux transporters to remove excess Cu and reduce the oxidative stress damage it causes. Also activated are genes involved in protein ubiquitination and degradation, probably to remove proteins damaged by Cu-induced oxidative stress. At the same time, Cu uptake transporters (CtrA1, CtrA2) are downregulated thereby presumably reducing Cu cellular uptake. Expression of genes participating in ribosome biogenesis and protein synthesis is strongly down-regulated, as these processes are energetically costly and need to be reduced under stress. Comparison between the transcriptional response of WT vs. *aceA* null reveals several interesting *aceA*-dependent gene groups (Figure 9a, right). The expression of *crpA* depends on the Cu-induced activation of *aceA*, validating previously published results [6], as does that of *cufA*, encoding a Cu-binding transcription factor of unknown function [6]. Several genes encoding proteins participating in iron uptake (FetD low affinity iron uptake transporter, FreB Cu-binding reductase involved in reductive iron uptake, Fre7 Cu-binding reductase of unknown function) are also activated in an *aceA*-dependent manner, as are three secondary metabolite clusters participating in the biosynthesis of the antibiotic triterpene helvolic acid [32], the prenylated indole alkaloid toxin fumitremorgin [33] and the antiparasitic angiogenesis inhibitor fumagillin [34], possibly as a general response to cellular stress. Cu-activated AceA strongly inhibits expression of the Cu chaperone AtxA (see below) and the secondary metabolite cluster synthesizing trypacidin, an anti-amoebic conidial toxin [35].

We selected six genes (*Afu7g06920* (*nmrA* redox sensor), *Afu4g00700* (*cipB*, oxidoreductase), *Afu3g07690 (cox17), Afu2g09700 (ccsA), Afu1g08880 (atxA)*, and *Afu3g14950* (*nirK*)), identified in the RNAseq analysis for further characterization, on the basis of their significant upregulation by Cu or their *aceA*-dependent activation. Deletion of *nmrA, cipB,* and *nirK* did not affect Cu-sensitivity. We focused on the analysis of the three *A. fumigatus* Cu chaperones encoded by *cox17, atxA* and *ccsA*.

### *A. fumigatus cox17* is an essential gene

In *S. cerevisiae*, Cox17 transfers Cu to the mitochondrial proteins Sco1 and Cox11 which insert it into CcO (cytochrome C oxidase) to support respiration. *S. cerevisiae cox17* null mutants are viable because of their ability to bypass respiration by fermentation [36]. Deletion of *cox17* in *A. fumigatus* resulted in greatly decreased hyphal growth rates, leading to the formation of tiny aconidial colonies which could not be propagated, suggesting that *cox17* is essential. Notably, the whole-genome deletion library of the filamentous fungus *Neurospora crassa* does not contain a *cox17* deletion mutant, indicating that it is also essential in this mold. To further understand the function of *cox17* in *A. fumigatus*, it will be necessary to generate a conditional mutant and analyze mitochondrial respiration rates and Cco1 levels and activity after a shift to repressive conditions.

### *A fumigatus* AtxA participates in reductive iron uptake

In *S. cerevisiae*, Atx1 transfers Cu to Ccc2, a P-type ATPase which imports Cu into the trans-golgi network where it is bound by Fet3 Cu-oxidase. Fet3 forms a complex with the high-affinity iron permease Ftr1 to enable reductive iron uptake. The *S. cerevisiae Δatx1* mutant is deficient in ferrous iron uptake and exhibits iron-dependent growth [9,37]. However, in *A. fumigatus*, unlike in *S. cerevisiae*, there are both reductive iron and siderophore-iron uptake pathways and deletion of *atxA* alone resulted in no obvious Fe or Cu dependent phenotype. To uncover the role of *A. fumigatus* AtxA in reductive iron uptake, we deleted both *atxA* and *sidA*, the first gene in the siderophore biosynthesis pathway. Under Cu starvation, the *ΔatxA/ΔsidA* double mutant was more sensitive to iron depletion compared with the *ΔsidA* and *ΔatxA* strains, suggesting that *A. fumigatus* AtxA participates in reductive iron uptake, possibly by supplying FetC Cu-oxidase with Cu. Importantly, the *A. fumigatus ΔatxA/ΔsidA* double mutant was less sensitive to iron starvation than the *ΔsidA/ΔftrA* strain, proposing that there could be additional proteins supplying Cu to FetC in the absence of AtxA. This of course, does not exclude the possibility that *A. fumigatus* AtxA also supplies Cu to other, as yet unknown, proteins.

Several questions are raised in this section of the study. (i) How is CtpA supplied with Cu? It is apparently not delivered by AtxA, as its deletion does not phenocopy that of CtpA. It is unlikely to be CcsA or Cox17 and there are no other chaperones encoded in the *A. fumigatus* genome. Perhaps CtpA binds free Cu directly from the cytosol. (ii) to which transporter does AtxA transfer Cu? A BlastP search with CtpA identifies only two related genes, unlikely to encode AtxA Cu acceptors- the *crpA* Cu exporter [5,6] and *pcaA* that transports cadmium and not Cu [38].

### CcsA is the Cu-chaperone of SOD1 in A. fumigatus

Our work highlights the important role of CcsA as a Cu-buffering agent, similar in its contribution to Sod1. In *S. cerevisiae* and *Candida albicans*, Ccs1 is required for delivery and insertion of Cu into Sod1 superoxide dismutase [27,39]. Here we provide evidence that supports a similar role for CcsA in *A. fumigatus* (i) *ccsA* deletion phenocopies the Cu and oxidative-stress sensitivity of *sod1* deletion. (ii) *ccsA* deletion results in loss of catalytic activity of *A. fumigatus* sod1. Interestingly, the *ΔccsA* mutant, despite being highly sensitive to oxidative stress and high levels of Cu, displayed normal WT virulence in a mouse model of lung infection. This agrees with previous findings demonstrating unaltered virulence after *A. fumigatus sod1* deletion and argues that CcsA does not activate additional Cu-activated proteins involved in virulence [17]. Possible explanations for the normal virulence of the *ΔccsA* mutant include the existence of additional mechanisms of oxidative stress resistance, such as ROS scavenging by glutathione and thioredoxin pathways, or ROS quenching by melanin and mannitol in conidia. Alternatively, ROS generation may not be the main mechanism involved in the immune response to *A. fumigatus* infection. It is also thought-provoking that while both the *ΔccsA* and *ΔcrpA* mutants show equally high Cu-sensitivity *in vitro, ΔccsA* retains full virulence in infected mice, whereas *ΔcrpA* shows significantly reduced virulence. A possible explanation for this apparent discrepancy is that CcsA primarily mitigates the oxidative stress induced by excess Cu, whereas CrpA, by exporting Cu from the cell, helps control additional aspects of Cu damage, such as metal co-factor displacement, that may directly affect virulence.

The roles of the *A. fumigatus* Cu chaperones, on the basis of this work, can be summarized as follows (Figure 9b): *cox17* is an essential gene with a possible but as yet unproven role in chaperoning Cu to Cco1 to enable mitochondrial respiration. AtxA takes part in reductive iron uptake probably by transferring Cu to an unknown ER-localized Cu transporter to activate FetC involved in reductive iron uptake. CcsA binds and probably directly transfers Cu to SOD1, allowing it to neutralize reactive oxygen species.

## Supplementary Material

Supplemental Material

## Data Availability

: The data that support the findings of this study are available from the corresponding author, [NO], upon reasonable request.
