# Supplementary material for: Transcriptional response of Aspergillus fumigatus to copper and the role of the Cu chaperones
Source: Virulence. 2021 Sep 1;12(1):2186–200. doi: 10.1080/21505594.2021.1958057 (PMC8425704; doi:10.1080/21505594.2021.1958057)
Supplement: Supplemental Material [file KVIR_A_1958057_SM5022.zip › downloadFromZipFile...pdf]

Supplementary Fig. S1. Scheme outlining gene deletion construct integration into the target locus by double homologous recombination at the 5’ and 3’ flanking sequences. The primer pairs used to verify gene deletion (primer pair G), and correct integration into the gene locus (5F and 3F primer pairs) are highlighted by the arrows.

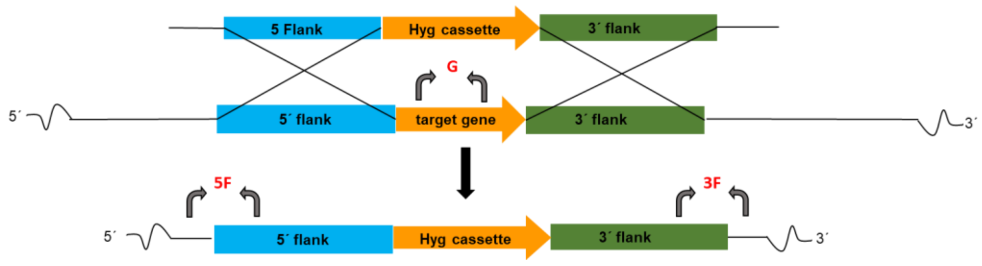

Supplementary Fig. S2. (A) Phenotype of three independent *Δcox17* isolates upon selection on YAG plates containing hygromycin (48 h, 37°C), indicating the small aconidial colonies. *ΔAfu7g0692*0 is provided as a positive, normal growth, control. (B) Colony radius of *Δcox17* grown in the absence of Cu (-Cu), or supplemented with a high concentration of Cu (10 µM Cu) for 48 h at 37°C on MMV plates.

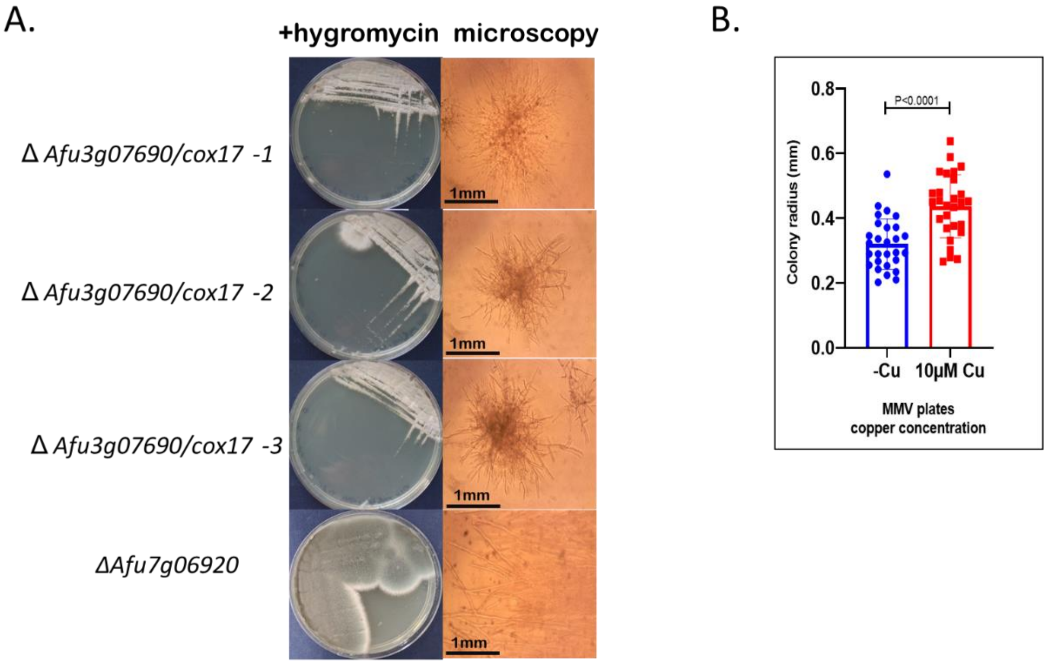

Table S1. ***A. fumigatus*** **strains used in this study.**

**Strain**	**Background strain**	**Genotype**	**Source**
akuB^{KU80} (WT)	*akuB*^{KU80} (*CEA17* background)	*ΔakuB::PyrG1*	Da Silva Ferreira, 2006
ΔnirK	*akuB*^{KU80}	*ΔakuB::PyrG1 ΔAFUA_3g14950::hph*	This study
ΔnmrA	*akuB*^{KU80}	*ΔakuB::PyrG1, ΔAFUA_7g06920::hph*	This study
ΔcipB	*akuB*^{KU80}	*ΔakuB::PyrG1, ΔAFUA_4g00700::hph*	This study
Δatx1	*akuB*^{KU80}	*ΔakuB::PyrG1, ΔAFUA_1g08880::hph*	This study
Δccs1	*akuB*^{KU80}	*ΔakuB::PyrG1, ΔAFUA_2g09700::hph*	This study
Δccs1-*rec (KI)*	*akuB*^{KU80}	*ΔakuB::PyrG1, ΔAFUA_2g09700::hph,AFUA_2g09700::phleo*	This study
Δcox17	*akuB*^{KU80}	*ΔakuB::PyrG1, ΔAFUA_3g07690*::*hph*	This study
ΔaceA	*akuB*^{KU80}	*ΔakuB::PyrG1, ΔAFUB_073740::PyrG*	Weimann, 2017
ΔcrpA	*akuB*^{KU80}	*ΔakuB::PyrG1, ΔAFUA_3g12740::PyrG*	Weimann, 2017
Δsod1	*akuB*^{KU80}	*ΔakuB::PyrG1, ΔAFUA_5g09240:: hph*	Lambou, 2010
Δsod2	*akuB*^{KU80}	*ΔakuB::PyrG1 ΔAFUA_4g11580:: hph*	Lambou, 2010
Δsod123	*akuB*^{KU80}	*ΔakuB::PyrG1 Δsod1::BLE/Δsod2::PTRa/Δsod3:: hph*	Lambou, 2010
ΔctpA	*CEA17*	*pyrG1, ΔctpA::pyrG+*	Upadhyay, 2013

ΔsidA	*CEA17*	*sidA::pyrG1*	Schrettl, 2004
ΔsidA/ΔftrA	*CEA17*	*sidA::pyrG1, ftrA::hph*	Schrettl, 2004
ΔsidA/Δatx1	*CEA17*	*sidA::pyrG1, ΔAFUA_1g08880::hph*	This study

Assembly of the construct for deletion of *cox17/Afu3g07690*. Primers were designed on NEBuilder (Table S2). The pGEM3 vector and *hph* selectable marker were amplified by PCR with 07690_Vector_Fwd /07690_Vector_Rev and 07690_Hyg_Fwd / 07690_Hyg_Rev primer pairs respectively using a plasmid template. 5' and 3' flanks of *Afu3g07690* were amplified with 07690_5Flank_Fwd/07690_5Flank_Rev and 07690_3Flank_Fwd /07690_3Flank_Rev respectively using WT genomic DNA. PCR was performed using the Phusion high fidelity DNA polymerse (Thermofisher). PCR products were verified for size on an agarose gel and purified with the Wizard SV gel and PCR cleanup kit (Promega). The resulting four fragments were ligated by GIBSON assembly using the NEBuilder Hifi DNA kit (NEB). Before transformation, the plasmid was linearized with *Asc*I. Verification of correct integration into the *Afu3g07690* locus in the transformants was not possible because they did not generate sufficient mycelium for genomic DNA preparation.

Table S2. Primers used for *cox17/Afu3g07690* deletion.

Name	Sequence 5'-3'
07690_Vector_fwd	ggcgcgccGACGTCGGGCCCAATTCG
07690_Vector_rev	ggcgcgccGCCTGCAGGTCGACCATATG
07690_5Flank_fwd	tctcccatatggtcgacctgcaggcggcgcgccGGGTGGCGGCTTTCGTCA
07690_5Flank_rev	ttctgtacctaggGGCGGATTTATGTTTTCTGGGTG
07690_Hyg_fwd	acataaatccgccCCTAGGTACAGAAGTCCAATTG
07690_Hyg_rev	cgactccaccagcTCTAGAAAGAAGGATTACCTCTAAAC
07690_3Flank_fwd	ccttctttctagaGCTGGTGGAGTCGATTGAAC
07690_3Flank_rev	cactgggcgaattgggcccgacgtcggcgcgccGAGAGGAAATATGCACTCGTC

Assembly of the construct for deletion of *nmrA/(Afu7g06920)*. Primers were designed on NEBuilder (Table S3). The pGEM3 vector and *hph* selectable marker were amplified by PCR with vector_6920_rev/vector_6920_fwd and hyg_69290_fwd/ hyg_6929 _rev primer pairs respectively using a plasmid template. 5' and 3' flanks of *Afu7g06920* were amplified with 5fl_6920_fwd/5fl_6920_rev and 3Fl_6920_fwd/3Fl_6920_rev respectively using WT genomic DNA. PCR was performed using the Phusion high fidelity DNA polymerse (Thermofisher). PCR products were verified for size on an agarose

gel and purified with the Wizard SV gel and PCR cleanup kit (Promega). The resulting four fragments were ligated by GIBSON assembly using the NEBuilder Hifi DNA kit (NEB). Before transformation, the plasmid was linearized with *Asc*I. Verification of correct integration into the *Afu7g06920* locus in the transformants was performed with the three primer pairs del06920G_FWD/ del06920G_REV, del06920G_5Fl_FWD/ del06920G_5Fl_REV and del06920G_3_Fl_FWD/ del06920G_3_Fl_REV.

Table S3. Primers used for *nmrA/(Afu7g06920)* deletion.

del06920G_FWD	TCTGTCATCCGCACAATCC
del06920G_REV	GATCCCTGCCGTTGAAGTAA
del06920G_5Fl_FWD	TTATGCTTCCGGCTCGTATG
del06920G_5Fl_REV	GCAGGAGAGGCACGATATTT
del06920G_3_Fl_FWD	AATGCTCCGTAACACCCAATAC
del06920G_3_Fl_REV	GCGTCCATTCGCCATTCA
vector_6920_rev	ggcgcgccGGCCGCCTGCAGGTCGAC
vector_6920_fwd	ggcgcgccAGTGAGTCGTATTACAATTCACTG
5fl_6920_fwd	ccatatggtcgacctgcaggcggccggcgcgccTGTTTCCACCATATTATGTG
5fl_6920_rev	ttctgtacctaggTTTGATGAATTGATAGCTATGATAG
hyg_69290_fwd	tcaattcatcaaaCCTAGGTACAGAAGTCCAATTG
hyg_6929 _rev	ctcacctcactcaTCTAGAAAGAAGGATTACCTCTAAAC
3Fl_6920_fwd	ccttctttctagaTGAGTGAGGTGAGCTTCCAATGGTAGTG
3Fl_6920_rev	ccagtgaattgtaatacgactcactggcgcgccGGTCCGTGGCTGCCGTGC

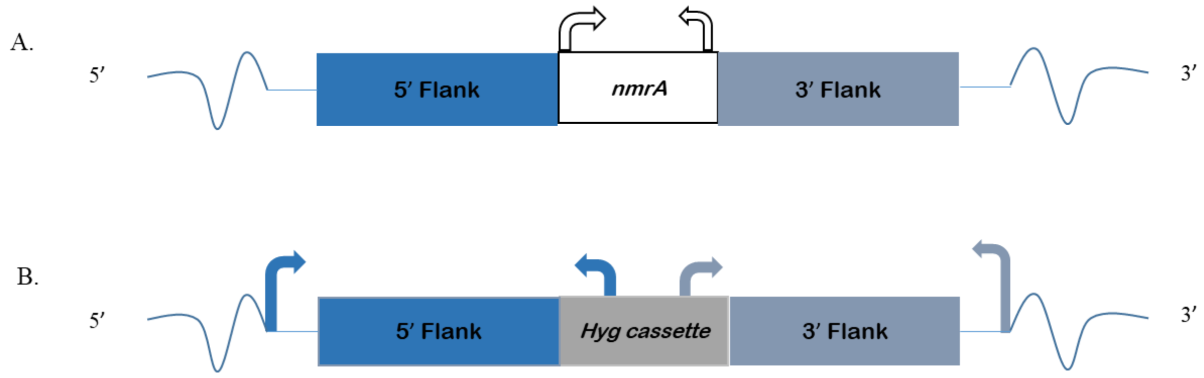

C.

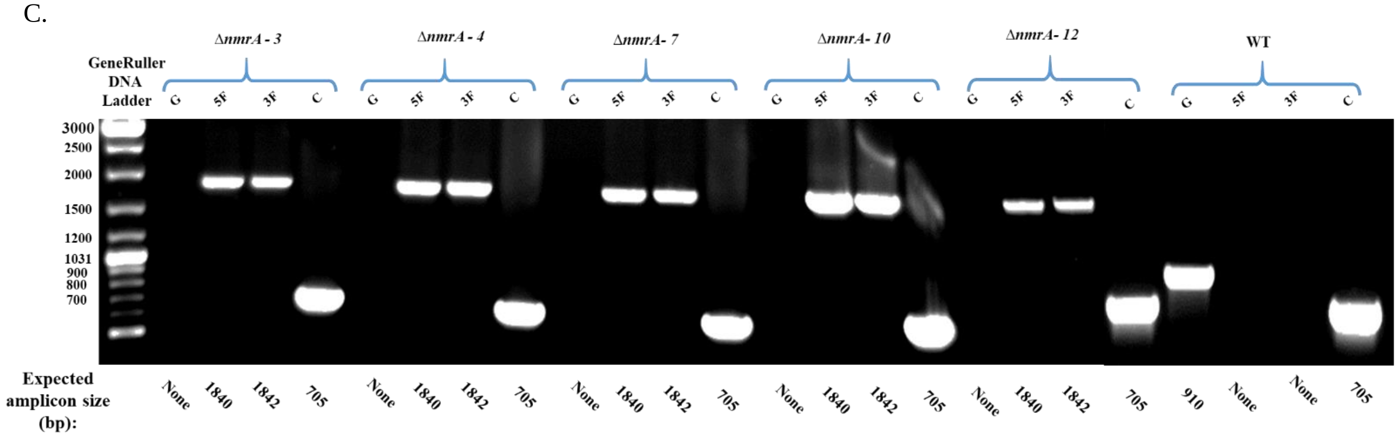

Figure S3. **PCR verification of *nmrA* gene deletion in *A. fumigatus***. **A.** The *ΔnmrA* gene with the 5' and 3' flanks before the transformation (WT). **B.** The hygromycin cassette with the homologous flanks after transformation generating the *ΔnmrA* null mutants (*ΔnmrA-3, ΔnmrA-4, ΔnmrA-7, ΔnmrA-10, ΔnmrA-12*), alongside the primers for the gene (white) and each one of the flanks; blue 5' flank and grey 3' flank, respectively. **C.** Ethidium bromide-stained agarose gel showing PCR products of the *nmrA* gene (G), 5'flank (5F), 3'flank (3F) and the *crpA gene* as a control (C) using colony DNA as PCR template.

Assembly of the construct for deletion of *cipB/Afu4g00700*. Primers were designed on NEBuilder (Table S4). The pGEM3 vector and *hph* selectable marker were amplified by PCR with 00700_Vector_fwd /00700_Vector_rev and 00700_Hyg_cassette_fwd / 00700_Hyg_cassette_rev primer pairs respectively using a plasmid template. 5' and 3' flanks of *Afu4g00700* were amplified with 00700_5_Flank_fwd /00700_5_Flank_rev and 00700_3_Flank_fwd /00700_3_Flank_rev _rev respectively using WT genomic DNA. PCR was performed using the Phusion high fidelity DNA polymerse (Thermofisher). PCR products were verified for size on an agarose gel and purified with the Wizard SV gel and PCR cleanup kit (Promega). The resulting four fragments were ligated by GIBSON assembly using the NEBuilder Hifi DNA kit (NEB). Before transformation, the plasmid was linearized with *Asc*I. Verification of correct integration into the *Afu4g00700* locus in the transformants was performed with the three primer pairs del00700G_FW / del00700G_REV, del00700_5F_FW / del00700_5F_REV and del00700_3F_FW / del00700_3F_REV.

Table S4. Primers used for *cipB/Afu4g00700* deletion.

Name	Sequence 5'-3'
00700_Vector_fwd	GGCGCGCCAGTGAGTCGTATTACAATTCACTG
00700_Vector_rev	GGCGCGCCGGCCGCCTGCAGGTCGAC

00700_5_Flank_fwd	CCATATGGTCGACCTGCAGGCGGCCGGCGCGCCCGCTGTTCTTCGAG CTCG
00700_5_Flank_rev	TTCTGTACCTAGGCAGCCGTATTTATATCTGAATCAC
00700_Hyg_cassette_fwd	ATAAATACGGCTGCCTAGGTACAGAAGTCCAATTG
00700_Hyg_cassette_rev	CACATTCAACAGGTCTAGAAAGAAGGATTACCTCTAAAC
00700_3_Flank_fwd	CCTTCTTTCTAGACCTGTTGAATGTGTCTTATG
00700_3_Flank_rev	CCAGTGAATTGTAATACGACTCACTGGCGCGCCTATCCTTGAGCCTGA GAATG
del00700G_FW	GCACTCGGTTGTTCCCTATT
del00700G_REV	ACTCCTTGTACACGGCTTTC
del00700_5F_FW	GCTCGTATGTTGTGTGGAATTG
del00700_5F_REV	GCAGGAGAGGCACGATATTT
del00700_3F_FW	AATGCTCCGTAACACCCAATAC
del00700_3F_REV	GCTTAATGCGCCGCTACA

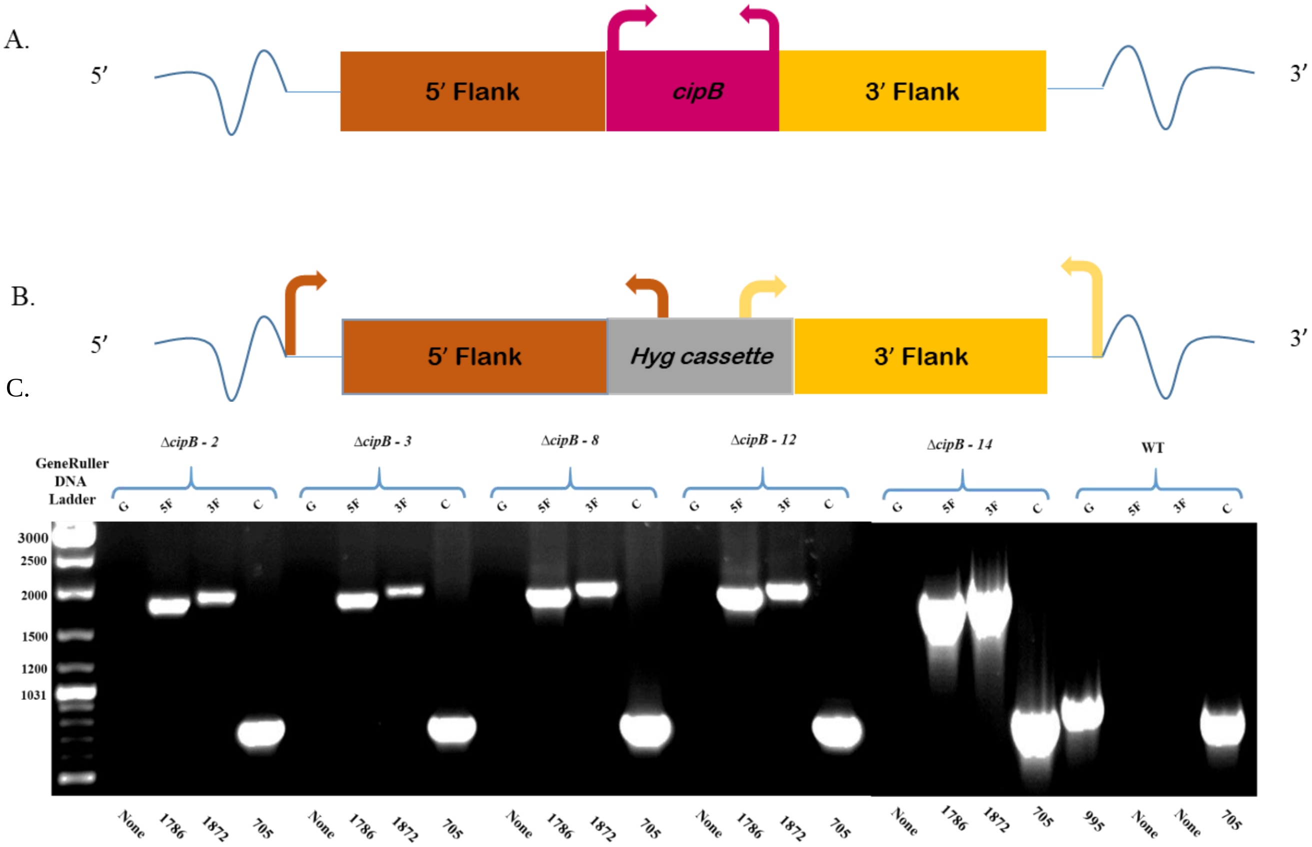

Figure S4. **PCR verification of *cipB* deletion in *A. fumigatus***. **A.** The *cipB* gene with the 5' and 3' flanks before transformation (WT). **B.** the hygromycin cassette with the homologous flanks after transformation generating the *ΔcipB* null mutants (*ΔcipB-2, ΔcipB-3, ΔcipB-8, ΔcipB-12, ΔcipB-14*),

alongside the primers for the gene (purple) and each one of the flanks; brown 5' flank and yellow 3' flank, respectively. **C.** Ethidium bromide-stained agarose gel showing PCR products of the *cipB* gene (G), 5'flank (5F), 3'flank (3F) and the *crpA gene* as a control (C) using colony DNA as PCR template.

Assembly of the construct for deletion of *ccs1/Afu2g09700*. Primers were designed on NEBuilder (Table S5). The pGEM3 vector and *hph* selectable marker were amplified by PCR with 09700_Vector_Fwd /09700_Vector_Rev and 09700_Hyg_Fwd / 09700_Hyg_Rev primer pairs respectively using a plasmid template. 5' and 3' flanks of *Afu2g09700* were amplified with 09700_5Flank_Fwd/09700_5Flank_Rev and 09700_3Flank_Fwd /09700_3Flank_Rev respectively using WT genomic DNA. PCR was performed using the Phusion high fidelity DNA polymerse (Thermofisher). PCR products were verified for size on an agarose gel and purified with the Wizard SV gel and PCR cleanup kit (Promega). The resulting four fragments were ligated by GIBSON assembly using the NEBuilder Hifi DNA kit (NEB). Before transformation, the plasmid was linearized with *Asc*I. Verification of correct integration into the *Afu2g09700* locus in the transformants was performed with the three primer pairs 09700_ColonyG_Fwd / 09700_ColonyG_Rev, 09700_Colony5F_Fwd / 09700_Colony5F_Rev and 09700_Colony3F_Fwd / 09700_Colony3F_Rev.

Table S5. Primers used for *ccs1/Afu2g09700* deletion.

Name	Sequence 5'-3'
09700_Vector_Rev	GGCGCGCCGGCCGCCTGCAGGTCGAC
09700_Vector_Fwd	GGCGCGCCGACGTCGGGCCCAATTCG
09700_5Flank_Fwd	CCATATGGTCGACCTGCAGGCGGCCGGCGCGCCGGCGGCGGATCGCCGTAG
09700_5Flank_Rev	TTCTGTACCTAGGTTCGGTATGACGCTAAAAGCTCCAGAAAATATATC
09700_Hyg_Fwd	GCGTCATACCGAACCTAGGTACAGAAGTCCAATTG
09700_Hyg_Rev	AGACACCAAAAGTTCTAGAAAGAAGGATTACCTCTAAAC
09700_3Flank_Fwd	CCTTCTTTCTAGAACTTTTGGTGTCTAGTCCGTCTTCCG
09700_3Flank_Rev	CACTGGGCGAATTGGGCCCGACGTCGGCGCGCCAGCCGTAAGCGCCCAGCG
09700_ColonyG_Fwd	GCGATCGATTACTGACTCCATT
09700_ColonyG_Rev	CGCTCCTGCCATACATTCTT
09700_Colony5F_Fwd	TGCCAGTAAAGACGACAGAAG
09700_Colony5F_Rev	GCAGGAGAGGCACGATATTT
09700_Colony3F_Fwd	CCCTGGGTTCGCAAAGATAA
09700_Colony3F_Rev	CGGAGATGAGCACAGAGATTG
CrpA_Fwd	ATGGCTACGGAAACGAGGCC
CrpA_Rev	CACCTCACGGACAAAGTCGAGC

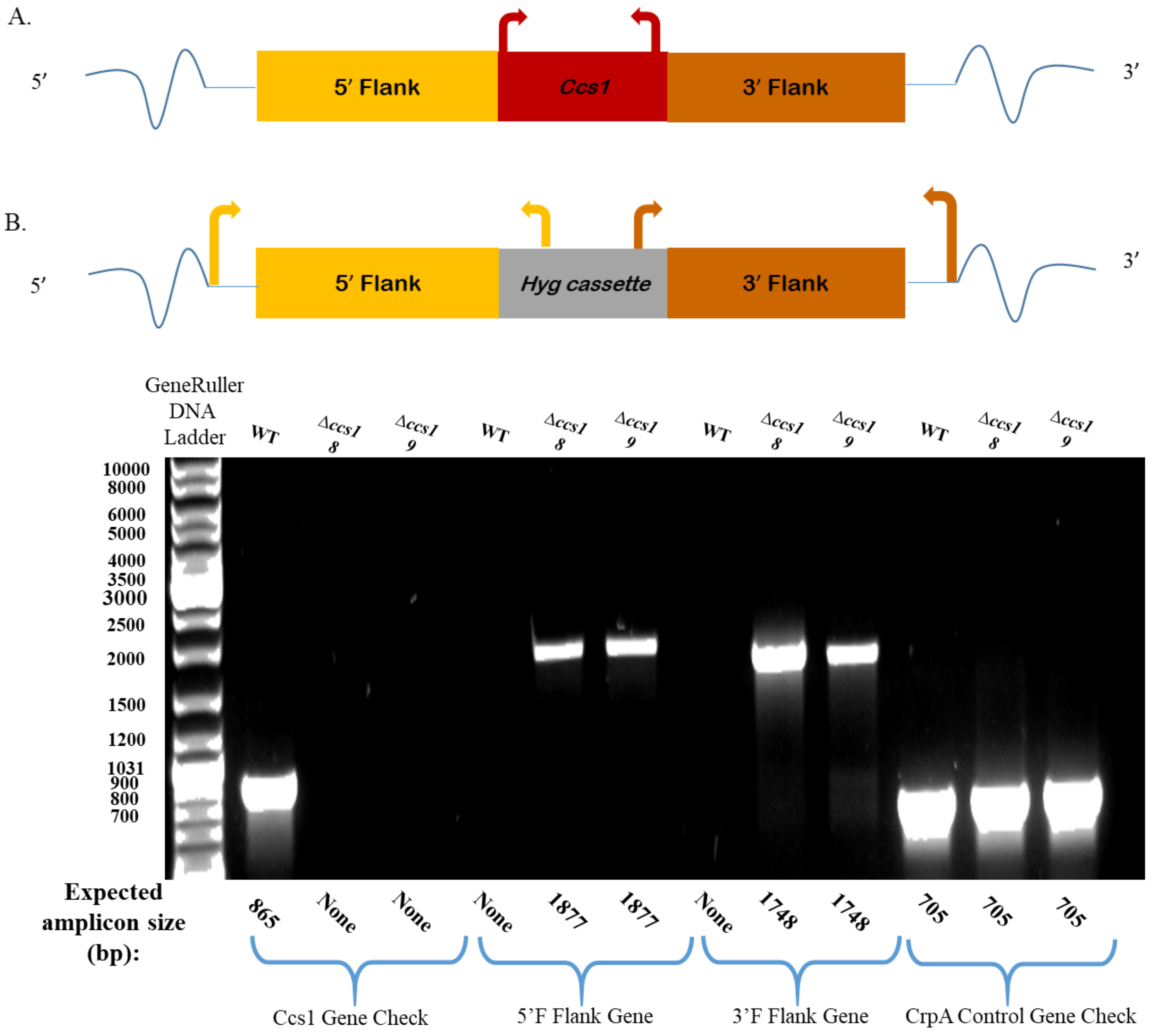

Figure S5. **PCR verification of *ccs1* deletion in *A. fumigatus*.** **A.** The *ccs1* gene with the 5’ and 3’ flanks before the transformation (WT). **B.** the hygromycin cassette with the homologous flanks after generating the *Δccs1* null mutants (*Δccs1-8, Δccs1-9*), alongside the primers for the gene (red) and each one of the flanks; yellow 5’ flank and brown 3’ flank, respectively. **C.** Ethidium bromide-stained agarose gel showing PCR products of the *ccs1* gene (G), 5’flank (5F), 3’flank (3F) and the *crpA gene* as a control (C) using colony DNA as PCR template.

Table S6. Primers used for *ccs1/Afu2g09700* reconstitution (KI)

Name	Sequence 5'-3'
Ccs1KIp_5FG3F_fwd	aatccttcttACTCCACATGTCGATTTCC

Ccs1KIp_5FG3F_rev	ggcgaattgggcccgacgtcATCGGAGATGAGCACAGAG
Ccs1KIp_phleo_fwd	tggtcgacctgcaggcggccGAATTCCCTTGTATCTCTACAC
Ccs1KIp_phleo_rev	catgtggagtAAGAAGGATTACCTCTAAACAAG
Ccs1KIp_vector_fwd	GACGTCGGGCCCAATTCGC
Ccs1KIp_vector_rev	GGCCGCCTGCAGGTCGAC

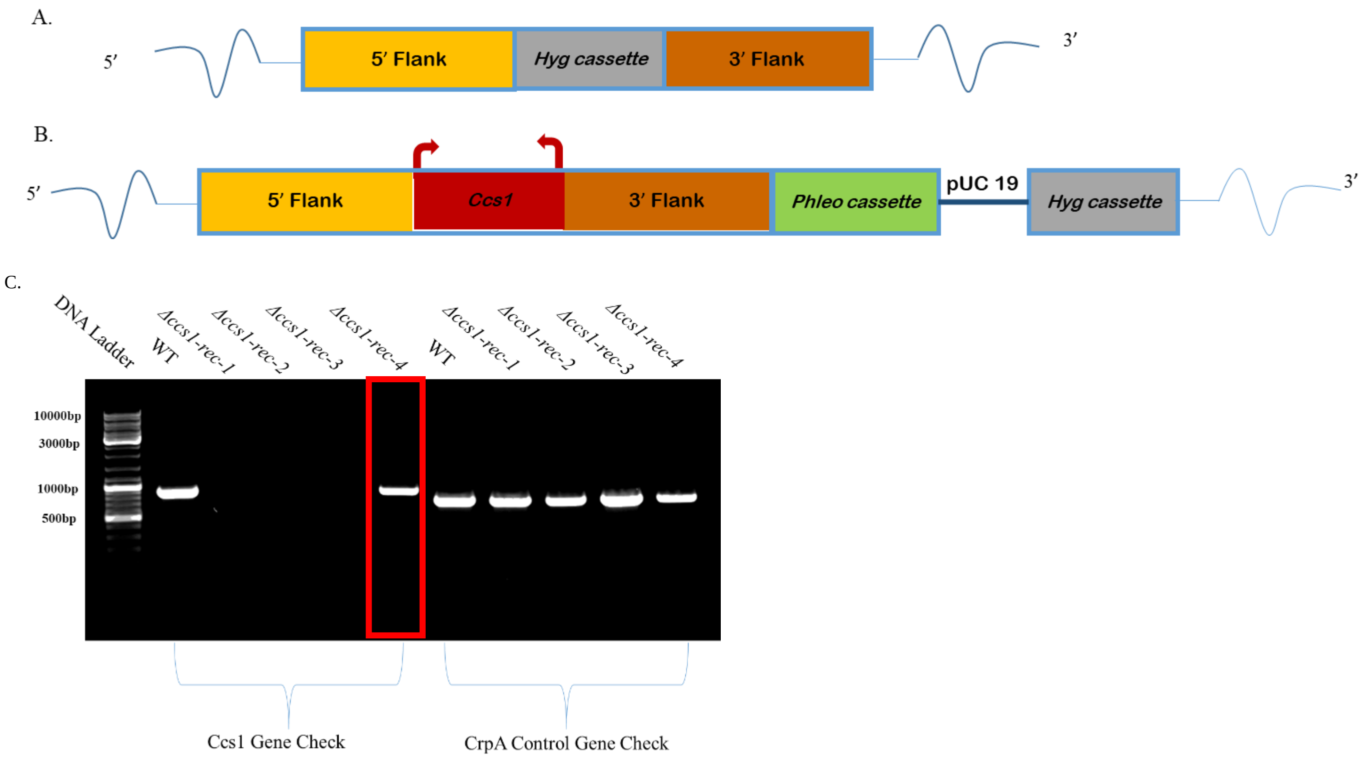

Figure S6. **PCR verification of the reconstitution of *ccs1* into *Δccs1*. A.** The hygromycin cassette containing locus with the 5' and 3' flanks before the transformation (*Δccs1*). **B.** The *ccs1* locus with the flanks after the transformation as expected in the *Δccs1-rec* null mutants (*Δccs1-rec1-4*), alongside the primers for the gene (red) and each one of the flanks; yellow 5' flank and brown 3' flank, and green phleo cassette, and blue pUC 19 vector, and grey Hyg cassette, respectively. **C.** Ethidium bromide-stained agarose gel showing PCR products of the *ccs1* gene (left) and the *crpA* gene (right) as a control using colony DNA as PCR template. Only isolate *Δccs1-rec-4* (red box, left) is positive for *ccs1*. The hyg should be there, because be only inserted the ccs1 gene with the phleo as a result the mutants are both hyg and phleo resistant and we checked the KI mutants on hyg as one of the controls.

Assembly of the construct for deletion of *atx1/Afu1g08880*. Primers were designed on NEBuilder (Table S7). The pGEM3 vector and *hph* selectable marker were amplified by PCR with 08880_Vector_Fwd /08880_Vector_Rev and 08880_Hyg_Cassette_Fwd / 08880_Hyg_Cassette_Rev primer pairs respectively using a plasmid template. 5' and 3' flanks of *Afu1g08880* were amplified with 08880_5Flank_Fwd /08880_5Flank_Rev and 08880_3Flank_Fwd/08880_3Flank_Rev respectively using WT genomic DNA. PCR was performed using the Phusion high fidelity DNA polymerse (Thermofisher). PCR products were verified for size on an agarose gel and purified with the Wizard SV gel and PCR cleanup kit (Promega). The resulting four fragments were ligated by GIBSON assembly using the NEBuilder Hifi DNA kit (NEB). Before transformation, the plasmid was linearized with *Asc*I. Verification of correct integration into the *Afu1g08880* locus in the transformants was performed with the three primer pairs 08880_ColonyG_Fwd / 08880_ColonyG_Rev, 08880_Colony5F_Fwd / 08880_Colony5F_Rev and 08880_Colony3F_Fwd/ 08880_Colony3F_Rev.

Table S7. Primers used for *atx1/Afu1g08880* deletion.

Name	Sequence 5'-3'
08880_Vector_Rev	GGCGCGCCGCCTGCAGGTCGACCATATG
08880_Vector_Fwd	GGCGCGCCGACGTCGGGCCCAATTCG
08880_5Flank_Fwd	TCTCCCATATGGTCGACCTGCAGGCGGCGCGCCACAGTCTGTCCCCCACTG
08880_5Flank_Rev	TTCTGTACCTAGGGGTGTACGAAGAACCAGTG
08880_Hyg_Cassette_Fwd	TTCTTCGTACACCCCTAGGTACAGAAGTCCAATTG
08880_Hyg_Cassette_Rev	CGCTGCACCAAAATCTAGAAAGAAGGATTACCTCTAAAC
08880_3Flank_Fwd	CCTTCTTTCTAGATTTTGGTGCAGCGCTCGTC
08880_3Flank_Rev	CACTGGGCGAATTGGGCCCGACGTCGGCGCGCCCAAATCCCCTTATCTTGCAGAGC
08880_ColonyG_Fwd	AGCATCAGTACAAATTCAATGTCTC
08880_ColonyG_Rev	TTTCTTGATCGTTGCCAATACAG
08880_Colony5F_Fwd	CAGACTTGGCTCATCTTCCTAC
08880_Colony5F_Rev	GCAGGAGAGGCACGATATTT
08880_Colony3F_Fwd	ATGCTCCGTAACACCCAATAC
08880_Colony3F_Rev	CAATCACGACCCTGCTACTTAC
CrpA_Fwd	ATGGCTACGGAAACGAGGCC
CrpA_Rev	CACCTCACGGACAAAGTCGAGC

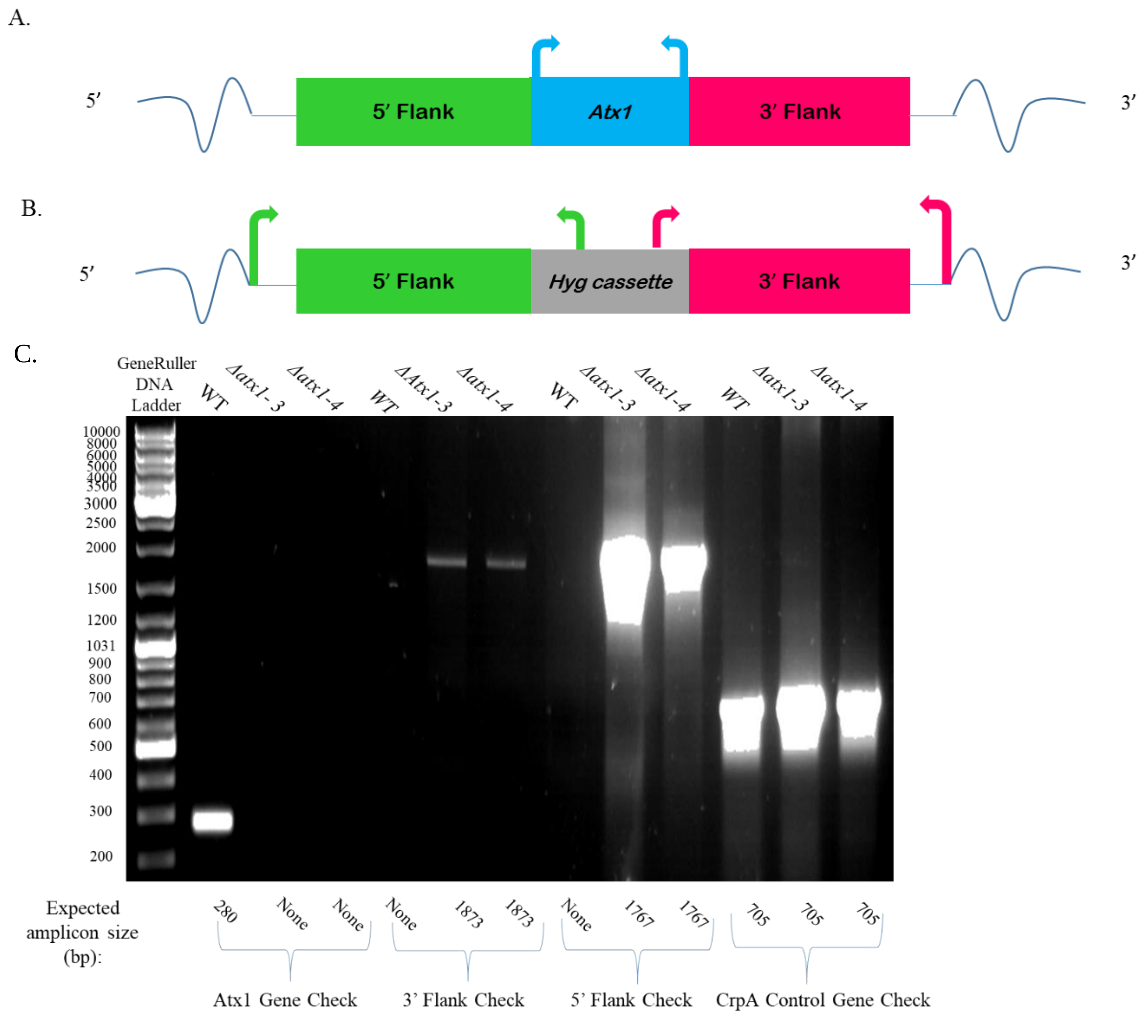

Figure S7. PCR verification of *atx1* deletion in *A. fumigatus*. **A.** The *atx1* gene with the 5' and 3' flanks before the transformation (WT). **B.** the hygromycin cassette with the homologous flanks after transformation, generating the *Δatx1* null mutants, alongside the primers for the gene (blue) and each one of the flanks; green 5' flank and pink 3' flank, respectively. **C.** Ethidium bromide-stained agarose gel showing PCR products of the *atx1* gene (G), 5'flank (5F), 3'flank (3F) and the *crpA gene* as a control (C) using colony DNA as PCR template.

A.

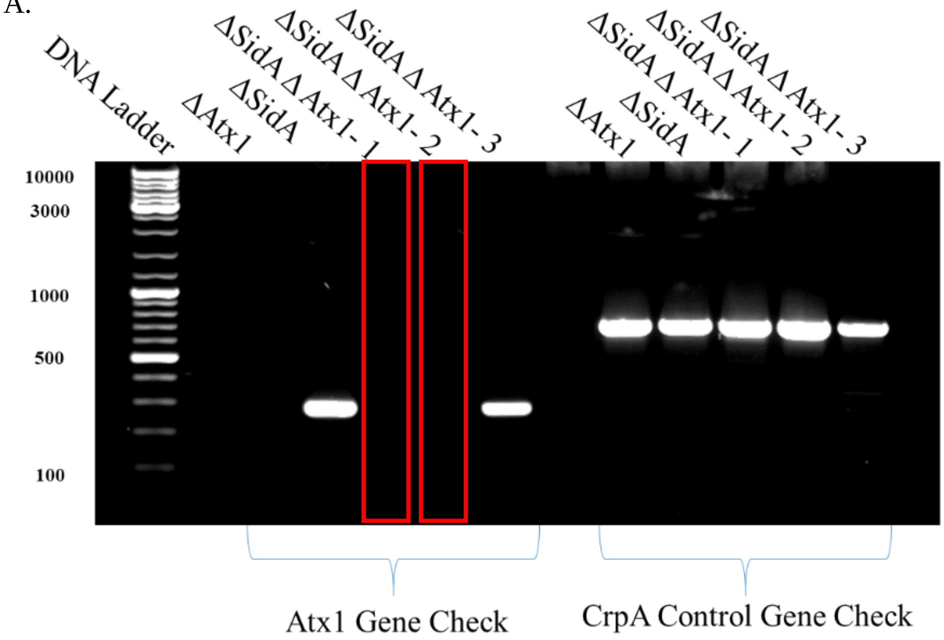

Fig S8. PCR verification of the *atx1* deletion in *ΔsidA* generating the *ΔsidA/Δatx1* double null strain.. Ethidium bromide-stained agarose gel showing PCR products of the *atx1* gene and the *crpA gene* as a control using colony DNA as PCR template. Red rectangles indicate deletion of *atx1* in the *ΔsidA* background.

Assembly of the construct for deletion of *nirK/Afu3g14950*. Primers were designed on NEBuilder (Table S8). The pGEM3 vector and *hph* selectable marker were amplified by PCR with 14950_Vector_Fwd /14950_Vector_Rev and 14950_Hyg_Cassette_Fwd / 14950_Hyg_Cassette_Rev primer pairs respectively using a plasmid template. 5’ and 3’ flanks of *Afu3g14950* were amplified with 14950_5Flank_Fwd /14950_5Flank_Rev and 14950_3Flank_Fwd /14950_3Flank_Rev respectively using WT genomic DNA. PCR was performed using the Phusion high fidelity DNA polymerse (Thermofisher). PCR products were verified for size on an agarose gel and purified with the Wizard SV gel and PCR cleanup kit (Promega). The resulting four fragments were ligated by GIBSON assembly using the NEBuilder Hifi DNA kit (NEB). Before transformation, the plasmid was linearized with *Asc*I. Verification of correct integration into the *Afu3g14950* locus in the transformants was performed with the three primer pairs 14950_ColonyG_Fwd / 14950_ColonyG_Rev, 14950_Colony5F_Fwd / 14950_Colony5F_Rev and 14950_Colony3F_Fwd / 14950_Colony3F_Rev.

Table S8. Primers used for *nirK* deletion.

14950_Vector_Rev	GGCGCGCCGCCTGCAGGTCGACCATATG
14950_Vector_Fwd	GGCGCGCCGACGTCGGGCCCAATTCG
14950_5Flank_Fwd	TCTCCCATATGGTCGACCTGCAGGCGGCGCGCCATCCAAGTTCGTGG GAGG
14950_5Flank_Rev	TTCTGTACCTAGGCCTGAGATTTTAACGTCAAAC
14950_Hyg_Cassette_F wd	TTAAAATCTCAGGCCTAGGTACAGAAGTCCAATTG
14950_Hyg_Cassette_R ev	CGACTTCTCAGATTCTAGAAAGAAGGATTACCTCTAAAC
14950_3Flank_Fwd	CCTTCTTTCTAGAATCTGAGAAGTCGGCTGTACGCATTG
14950_3Flank_Rev	CACTGGGCGAATTGGGCCCGACGTCGGCGCGCCCAGGCCATCCGCCT GCAT
14950_ColonyG_Fwd	CGTAGTCCAGCCCTGATAAA
14950_ColonyG_Rev	CCGCATTCCCAAAGAAGATG
14950_Colony5F_Fwd	GCCGTGTAGATGGAGGTAAAC
14950_Colony5F_Rev	CGATATTTGGACGCCCTACAG
14950_Colony3F_Fwd	GCCTACAGGACACACATTCAT
14950_Colony3F_Rev	CAGGTACTCCTTGGCATGTTAG

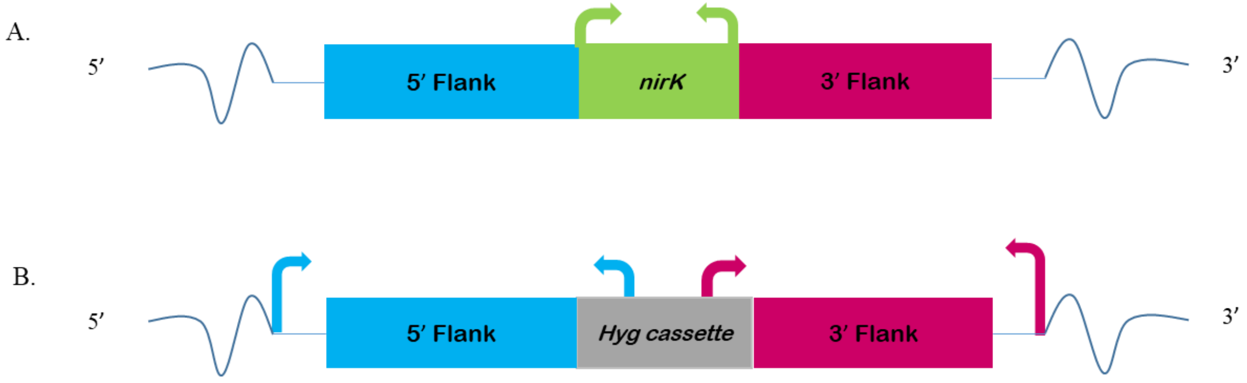

C.

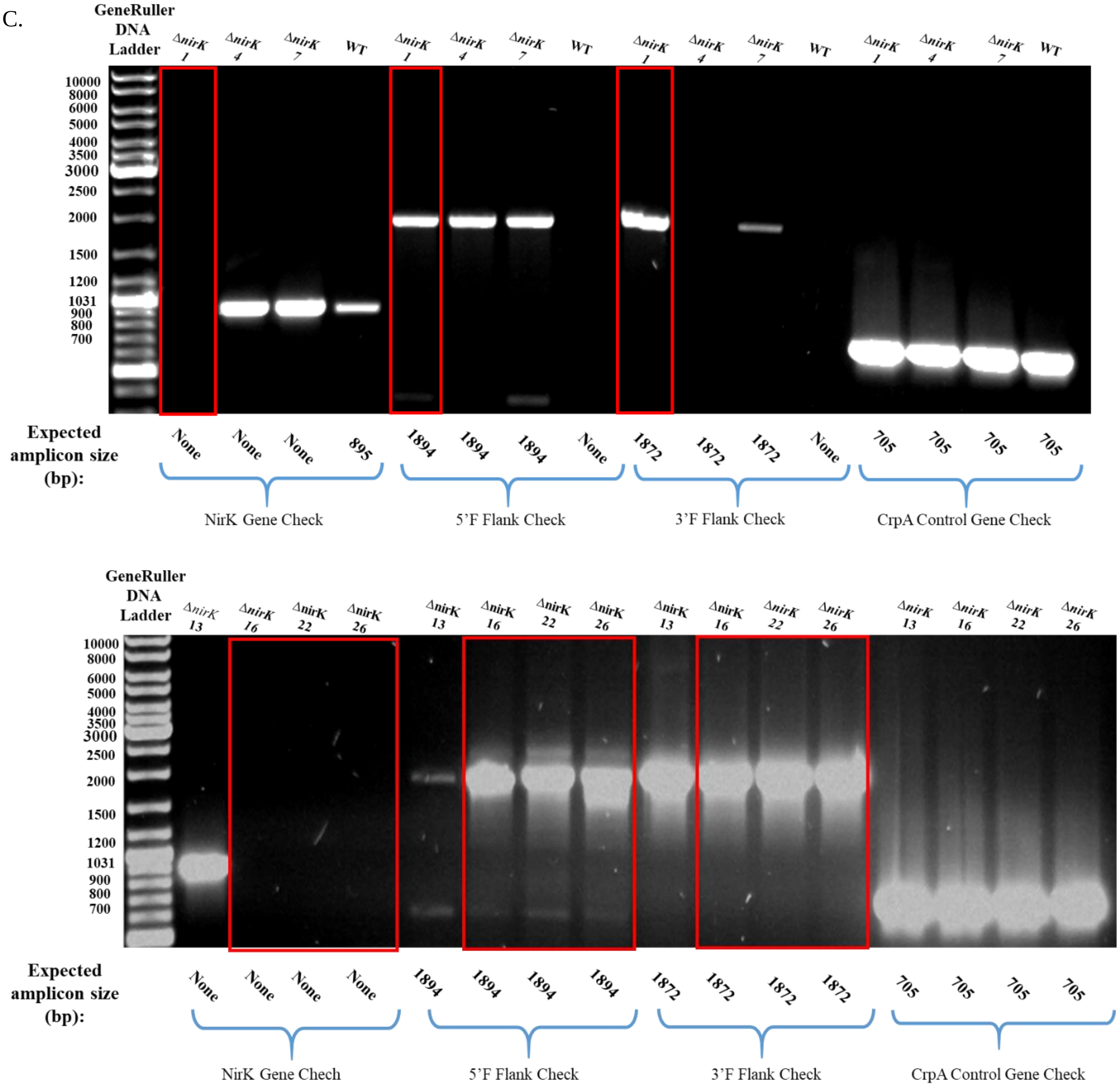

Figure S9. **PCR verification of *nirK* deletion in *A. fumigatus***. **A.** The *nirK* gene with 5' and 3' flanks before transformation (WT). **B.** the hygromycin cassette with the homologous flanks after generation of the *ΔnirK* null mutants (*ΔnirK-1, ΔnirK-16, ΔnirK-22, ΔnirK-26*), alongside the primers for the gene (green) and each one of the flanks; blue 5' flank and pink 3' flank, respectively. **C.** Ethidium bromide-stained agarose gel showing PCR products of the *nirK* gene (G), 5'flank (5F), 3'flank (3F) and the *crpA gene* as a control (C) using colony DNA as PCR template.

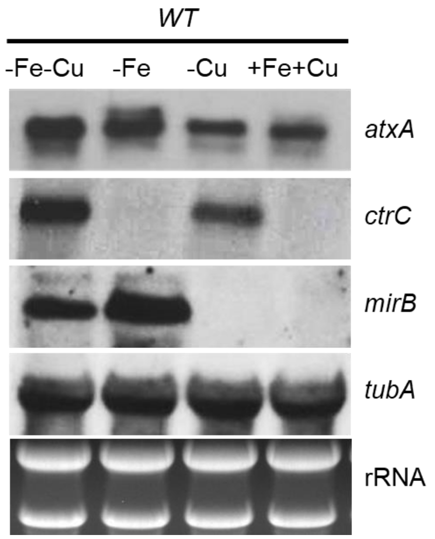

Figure S10. **Northern blot of *atxA*, *ctrC* and *mirB* gene expression**. Expression was tested in the WT strain under standard conditions (MM) or exposed to starvation for Cu (-Cu), Fe (-Fe) starvation or both (-Fe-Cu). *TubA* housekeeping gene expression level and ethidium bromide-stained ribosomal RNA are shown as control for RNA loading and quality.

Detailed description of qPCR gene expression analysis.

All qPCRs were performed using the primer pairs shown in Table S9 and SYBR Green as follows: 95°C for 20 sec (holding stage), and then 40 cycles of 95°C for 1 sec, 60°C for 20 sec (cycling stage), then 95°C for 15 sec, 60°C for 60 sec (melt curve stage) and hold at 4°C. The specificity of the reaction was verified by melt curve analysis. Ct values for technical replicates of each gene in both WT and Δ*AceA* in treated and untreated conditions were averaged (see Table below). Then, ΔCt was calculated as the ratio for each gene compared to the reference gene (β-tubulin). From the three biological replicates for each gene (WT/ Δ*AceA*; treated/untreated) the average ΔCt and standard deviation for each gene was calculated. The fold expression after Cu treatment was calculated as ΔΔCt (ΔCt of treated samples minus ΔCt of untreated samples). All values were transferred to Prism, mean value and standard deviations were calculated using 2-way Anova.

	b-tubulin		*Afu3g14950*		*Afu7g06929*		*Afu4g00700*		*Afu3g07690*		*Afu1g08880*		*Afu2g09700*	
WT -Cu #1	20.37756	20.02224	26.1554	25.90265	25.21819	25.23819	26.42865	26.58488	23.91031	24.40407	33.73754	34.1384	24.79547	24.85495
WT -Cu #2	20.53524	20.17953	26.10126	25.60184	24.78685	24.64637	25.67613	25.84494	24.08244	24.08742			24.81343	24.80698
WT -Cu #3	20.72615	20.55206	26.34827	26.17762	25.84352	25.9424	27.24048	27.3222	25.14428	25.16105	32.46754	32.25881	24.86837	24.83269
WT +Cu #4	21.8858	21.6245	26.02224	26.97579	19.69093	19.70052	22.75855	22.71581	22.23326	22.72121	33.16701	33.69475	23.21471	
WT +Cu #5	21.94256	21.75704	26.52973	26.46451	19.82277	19.78839	22.68873	22.66248	22.35162	22.71175	33.42142	34.04724	23.21556	23.05851
WT +Cu #6	21.84003	21.59889	26.38104	26.47476	19.95049	19.88321	22.95952	22.76026	22.6	22.93823	33.96153	33.4563	22.97272	23.13988
ΔAceA -CU #1	20.29737	19.90667	27.3645	27.32347	26.24043	26.19886	27.63095	27.76814	25.00162	25.57279			24.14092	24.32436
ΔAceA -Cu #2	20.13173	19.46385	25.6356	25.79316	25.01121	24.64775	26.34861	26.46097	24.54715		20.18431	20.26401	24.47488	24.67017
ΔAceA -CU #3	20.70895	20.11794	28.26485	28.46728	27.96211	27.74187	30.03974	28.75706		25.5682	20.1539	20.10495	24.69285	24.71759
ΔAceA +Cu #4	20.97198	20.47721	26.94003	27.72208	19.53777	19.18006	23.36699	23.31334	22.89072	22.72527	21.49591	21.5755	22.04917	22.11028
ΔAceA +Cu #5	21.51204	21.16109	27.52447	27.24492	19.83432	19.64016	23.85838	23.79034	23.08314	23.24846	22.05097	22.21617	22.82447	22.84178
ΔAceA +Cu #6	22.31067		26.15862	26.05388	20.62369	20.589	24.51263	24.46682	23.16057	23.02725	22.07774	21.80005	23.58589	23.70836

Table S9. Primers used in the qPCR analysis.

	Name	Sequence 5' – 3'
1	βtub-qPCR-F	GGTAACTCCACCTCCATTCAG
	βtub-qPCR-R	AACTCCATCTCGTCCATACC
2	Afu7g06920-F	TGTGCTGCCAGGGTATTT
	Afu7g06920-R	GTGGAAACTTGGCGTTCTG
3	Afu4g00700-F	CGACAGATGGGATTCAGGT
	Afu4g00700-R	TCAGGAGAAGGCAGAAAGG
4	Afu3g14950-F	CGGAGTGAGCCTGTTCTT
	Afu3g14950-R	GGGTAGGTCAATGTCGTCTT
5	Afu3g07690-F	TCTTCGGCTCTTCCAAAGG
	Afu3g07690-R	CGGACTTCTCGGTCTTACAG
6	Afu2g09700-F	ATTGAGCCGTTCCAGACC
	Afu2g09700-R	GATTCGCTTCCACCCTCTT
7	Afu1g08880-F	GTCTCCATGAGCTGTGGT
	Afu1g08880-R	CAGTGGGTTCTGTCGTGA

<u>Table S10:</u> Go annotation of upregulated **(A)** and downregulated **(B)** *A. fumigatus* WT genes in response to copper.

**A. Upregulated genes**	
**Category**	**Gene**
Modification-dependent protein catabolic process<br>n= 49<br>*P* = 0.00452	*Afu1g02830, Afu1g05040, Afu1g06420, Afu1g06850, Afu1g07555, Afu1g15920, Afu2g03400, Afu2g04720, Afu2g04740, Afu2g06330, Afu2g09240, Afu2g14110, Afu3g06610, Afu3g06700, Afu3g07820, Afu3g08940, Afu3g11390, Afu3g11700, Afu4g04100, Afu4g04280, Afu4g04640, Afu4g04660, Afu4g06090, Afu4g07340, Afu4g07420, Afu4g07520, Afu4g12910, Afu5g03610, Afu5g05705, Afu5g07050, Afu5g08370, Afu5g10740, Afu5g11720, Afu5g13890, Afu6g04750, Afu6g06350, Afu6g08220, Afu7g04650, Afu8g03960, cdc48, ddiA, hrdA, pre1, pre6, pre8, pre9, prs2, prs3, rpt4*
Ubiquitin-dependent protein catabolic process<br>n= 49<br>*P* = 0.00452	*Afu1g02830, Afu1g05040, Afu1g06420, Afu1g06850, Afu1g07555, Afu1g15920, Afu2g03400, Afu2g04720, Afu2g04740, Afu2g06330, Afu2g09240, Afu2g14110, Afu3g06610, Afu3g06700, Afu3g07820, Afu3g08940, Afu3g11390, Afu3g11700, Afu4g04100, Afu4g04280, Afu4g04640, Afu4g04660, Afu4g06090, Afu4g07340, Afu4g07420, Afu4g07520, Afu4g12910, Afu5g03610, Afu5g05705, Afu5g07050, Afu5g08370, Afu5g10740, Afu5g11720, Afu5g13890, Afu6g04750, Afu6g06350, Afu6g08220, Afu7g04650, Afu8g03960, cdc48, ddiA, hrdA, pre1, pre6, pre8, pre9, prs2, prs3, rpt4*
Modification-dependent macromolecular catabolic process<br>n= 50<br>*P* = 0.01679	*Afu1g02830, Afu1g05040, Afu1g06420, Afu1g06850, Afu1g07555, Afu1g13730, Afu1g15920, Afu2g03400, Afu2g04720, Afu2g04740, Afu2g06330, Afu2g09240, Afu2g14110, Afu3g06610, Afu3g06700, Afu3g07820, Afu3g08940, Afu3g11390, Afu3g11700, Afu4g04100, Afu4g04280, Afu4g04640, Afu4g04660, Afu4g06090, Afu4g07340, Afu4g07420, Afu4g07520, Afu4g12910, Afu5g03610, Afu5g05705, Afu5g07050, Afu5g08370, Afu5g10740, Afu5g11720, Afu5g13890, Afu6g04750, Afu6g06350, Afu6g08220, Afu7g04650, Afu8g03960, cdc48, ddiA, hrdA, pre1, pre6, pre8, pre9, prs2, prs3, rpt4*

**B. Downregulated genes**	
**Category**	**Gene**
Ribosome biogenesis<br>n= 102<br>*P* = 1.3E-17	*Afu1g03390, Afu1g04320, Afu1g04840, Afu1g05110, Afu1g05310, Afu1g05340, Afu1g05630, Afu1g06290, Afu1g06600, Afu1g06690, Afu1g06770, Afu1g10310, Afu1g10510, Afu1g10560, Afu1g10990, Afu1g11130, Afu1g12890, Afu1g13070, Afu1g13460, Afu1g13570, Afu1g13880, Afu1g14580, Afu1g15020, Afu1g16330, Afu1g16730, Afu2g01480, Afu2g01780, Afu2g02190, Afu2g02320, Afu2g03340, Afu2g03590, Afu2g03740, Afu2g04130, Afu2g05560, Afu2g05950, Afu2g08850, Afu2g10090, Afu2g10300, Afu2g10440, Afu2g10500, Afu2g11380, Afu2g11510, Afu2g12880, Afu2g12890, Afu2g13570, Afu2g17050, Afu3g06640, Afu3g06970, Afu3g08090, Afu3g09340, Afu3g10730, Afu3g11260, Afu3g12490, Afu4g03880, Afu4g05880, Afu4g07730, Afu4g08190, Afu4g08930, Afu4g10800, Afu4g13330,*

	Afu4g13690, Afu5g01760, Afu5g03090, Afu5g03870, Afu5g05630, Afu5g05710, Afu5g06010, Afu5g07020, Afu5g10840, Afu5g11150, Afu5g11620, Afu5g12100, Afu5g13470, Afu6g02270, Afu6g03580, Afu6g04260, Afu6g08900, Afu6g09820, Afu6g12660, Afu6g12920, Afu6g12990, Afu6g13550, Afu7g00870, Afu7g01460, Afu7g02140, Afu7g02210, Afu7g04490, Afu7g04860, Afu7g05290, Afu8g04220, Afu8g04790, S10a, hscA, lcp5, mrh4, nop5, nopA, rpL3, rps1, rps23, rsa4, ubiA
Ribonucleoprotein complex biogenesis<br>n= 105<br>*P* = 9.46E-14	*Afu1g03390, Afu1g04320, Afu1g04840, Afu1g05110, Afu1g05310, Afu1g05340, Afu1g05630, Afu1g06290, Afu1g06600, Afu1g06690, Afu1g06770, Afu1g10310, Afu1g10510, Afu1g10560, Afu1g10990, Afu1g11130, Afu1g12890, Afu1g13070, Afu1g13460, Afu1g13570, Afu1g13880, Afu1g14580, Afu1g15020, Afu1g16330, Afu1g16730, Afu2g01480, Afu2g01780, Afu2g01900, Afu2g02190, Afu2g02320, Afu2g03340, Afu2g03590, Afu2g03740, Afu2g04130, Afu2g05560, Afu2g05950, Afu2g08850, Afu2g10090, Afu2g10300, Afu2g10430, Afu2g10440, Afu2g10500, Afu2g11380, Afu2g11510, Afu2g12880, Afu2g12890, Afu2g13570, Afu2g17050, Afu3g06640, Afu3g06970, Afu3g08090, Afu3g09340, Afu3g10730, Afu3g11260, Afu3g12490, Afu4g03880, Afu4g05880, Afu4g07730, Afu4g08190, Afu4g08930, Afu4g10800, Afu4g13330, Afu4g13690, Afu5g01760, Afu5g03090, Afu5g03870, Afu5g05630, Afu5g05710, Afu5g06010, Afu5g07020, Afu5g10840, Afu5g11150, Afu5g11620, Afu5g12100, Afu5g13470, Afu6g02270, Afu6g02520, Afu6g03580, Afu6g04260, Afu6g08900, Afu6g09820, Afu6g12660, Afu6g12920, Afu6g12990, Afu6g13550, Afu7g00870, Afu7g01460, Afu7g02140, Afu7g02210, Afu7g04490, Afu7g04860, Afu7g05290, Afu8g04220, Afu8g04790, S10a, hscA, lcp5, mrh4, nop5, nopA, rpL3, rps1, rps23, rsa4, ubiA*
rRNA processing<br>n= 67<br>*P* = 1.62E-9	*Afu1g04320, Afu1g04840, Afu1g06290, Afu1g06690, Afu1g10510, Afu1g10560, Afu1g10990, Afu1g13070, Afu1g13460, Afu1g13570, Afu1g14580, Afu1g16730, Afu2g01780, Afu2g02190, Afu2g02320, Afu2g03340, Afu2g03590, Afu2g03740, Afu2g04130, Afu2g05560, Afu2g05950, Afu2g10440, Afu2g10500, Afu2g11380, Afu2g11510, Afu2g12890, Afu2g17050, Afu3g06640, Afu3g06970, Afu3g08090, Afu3g09340, Afu3g11260, Afu4g03880, Afu4g05880, Afu4g10800,* Afu4g13330, Afu5g01760, Afu5g03090, Afu5g03870, Afu5g05710, Afu5g06010, Afu5g10840, Afu5g11150, Afu5g11620, Afu5g12100, Afu5g13470, Afu6g02270, Afu6g04260, Afu6g08900, Afu6g09820, Afu6g12990, Afu6g13550, Afu7g00870, Afu7g02140, Afu7g02210, Afu7g04490, Afu7g04860, Afu7g05290, Afu8g04220, Afu8g04790, S10a, hscA, lcp5, nop5, nopA, rps1, rps23

<u>Table S11:</u> Go annotation of upregulated **(A)** and downregulated **(B)** *A. fumigatus* WT vs. Δ*aceA* strain in response to copper.

**A. Upregulated genes**	
**Category**	**Gene**
2nd metabolites<br>n= 19<br>*P*= 3.18E-5	*Afu3g02010, Afu4g14820, Afu7g04980, Afu8g00430, Afu8g00480, Afu8g00510, brlA, cyp5081A1, cyp5081B1, cyp5081C1, cyp5081D1, fqzB, ftmD, ftmF, ftmPT1, metAP, osc3, ppoC, sdr1*
Oxidation-reduction<br>n= 34<br>*P* = 0.00069	*Afu1g12210, Afu2g01160, Afu2g14430, Afu3g00800, Afu3g02010, Afu3g03180, Afu3g03930, Afu5g03540, Afu5g09720, Afu5g12470, Afu6g02170, Afu6g02820, Afu6g11020, Afu6g11850, Afu6g13350, Afu6g13945, Afu7g01000, Afu7g01450, Afu8g00510, Afu8g00560, Afu8g02560, FAOX-II, alcB, aldA, cyp5081A1, cyp5081B1, cyp5081C1, cyp5081D1, fqzB, fre2, ftmC, ftmE, glpV, ppoC*
Ether metabolic and biosynthetic process<br>n= 7<br>*P* = 0.00084	*Afu8g00430, Afu8g00480, Afu8g00510, Afu8g01090, ftmD, ftmF, metAP*
Metal ion transport<br>n= 9<br>*P* = 0.02673	*Afu2g08830, Afu3g12740, Afu6g02170, Afu6g02810, Afu6g02820, Afu6g11840, cch1, fre2, pmcA*
**B. Downregulated genes**	
**Category**	**Gene**
Neutral amino acid transport<br>n= 6<br>*P* = 0.00189	*Afu2g15140, Afu4g03220, Afu4g09040, Afu5g01510, Afu6g09110, Afu8g05860*
Transmembrane transport<br>n= 64<br>*P* = 0.02407	*Afu1g10370, Afu1g16750, Afu1g16880, Afu1g17160, Afu1g17570, Afu2g04550, Afu2g09282, Afu2g09570, Afu2g11520, Afu2g11580, Afu2g12080, Afu2g15140, Afu2g15410, Afu2g17660, Afu3g00200, Afu3g01840, Afu3g02080, Afu3g03320, Afu3g07950, Afu3g12170, Afu3g12720, Afu3g14170, Afu4g00550, Afu4g00570, Afu4g00990, Afu4g03220, Afu4g03390, Afu4g03740, Afu4g08800, Afu4g09210, Afu4g09410, Afu4g14670, Afu5g01510, Afu5g01630, Afu5g02390, Afu5g06210, Afu5g09940, Afu5g12950, Afu6g03080, Afu6g03720, Afu6g09110, Afu6g11100, Afu6g11320, Afu6g13780, Afu7g00390, Afu7g05100, Afu7g06120, Afu8g00770, Afu8g01180, Afu8g01480, Afu8g02200, Afu8g04110, Afu8g05860, fmqE, hxt8, mfs56, nrtB, optC, optD, optG, optH, ptr2, ssu1, zrfB*
Amide transport<br>n= 8<br>*P* = 0.06639	*Afu1g16880, Afu2g15140, Afu4g08800, Afu5g01630, Afu6g09110, optD, optG, ptr2*
